# Alveolar macrophage metabolic programming via a C-type lectin receptor protects against lipo-toxicity and cell death

**DOI:** 10.1038/s41467-022-34935-w

**Published:** 2022-11-25

**Authors:** Michal Scur, Ahmad Bakur Mahmoud, Sayanti Dey, Farah Abdalbarri, Iona Stylianides, Daniel Medina-Luna, Gayani S. Gamage, Aaron Woblistin, Alexa N. M. Wilson, Haggag S. Zein, Ashley Stueck, Andrew Wight, Oscar A. Aguilar, Francesca Di Cara, Brendon D. Parsons, Mir Munir A. Rahim, James R. Carlyle, Andrew P. Makrigiannis

**Affiliations:** 1 Department of Microbiology and Immunology, Dalhousie University, Halifax, NS Canada; 2 College of Applied Medical Science, Taibah University, Madina, Saudi Arabia; 3 Department of Pathology, McGill University, Montreal, QC Canada; 4 Beatrice Hunter Cancer Research Institute, Halifax, NS Canada; 5 Department of Biochemistry, Dalhousie University, Halifax, NS Canada; 6 Immunology Research Program, King Abdullah International Medical Research Centre, Riyadh, Saudi Arabia; 7 Department of Pathology, Dalhousie University, Halifax, NS Canada; 8 Department of Immunology, Dana-Farber Cancer Institute, Boston, MA USA; 9 Department of Microbiology and Immunology, University of California, San Francisco, USA; 10 Department of Biomedical Sciences, University of Windsor, Windsor, ON Canada; 11 Department of Immunology, University of Toronto, Toronto, ON Canada

**Keywords:** Alveolar macrophages, Mucosal immunology, Infection, Innate immunity

## Abstract

Alveolar macrophages (AM) hold lung homeostasis intact. In addition to the defense against inhaled pathogens and deleterious inflammation, AM also maintain pulmonary surfactant homeostasis, a vital lung function that prevents pulmonary alveolar proteinosis. Signals transmitted between AM and pneumocytes of the pulmonary niche coordinate these specialized functions. However, the mechanisms that guide the metabolic homeostasis of AM remain largely elusive. We show that the NK cell-associated receptor, NKR-P1B, is expressed by AM and is essential for metabolic programming. *Nkrp1b*^−/−^ mice are vulnerable to pneumococcal infection due to an age-dependent collapse in the number of AM and the formation of lipid-laden AM. The AM of *Nkrp1b*^−/−^ mice show increased uptake but defective metabolism of surfactant lipids. We identify a physical relay between AM and alveolar type-II pneumocytes that is dependent on pneumocyte Clr-g expression. These findings implicate the NKR-P1B:Clr-g signaling axis in AM-pneumocyte communication as being important for maintaining metabolism in AM.

## Abstract (web summary)

Alveolar macrophages (AM) in the lungs maintain surfactant during homeostasis and respond to infectious pathogens. Here the authors show that in the absence of NKR-P1B, pneumococcal infection is more severe because KO AM have increased rates of lipid surfactant uptake and reduced anti-microbial function.

## Introduction

The innate component of the mammalian immune system is composed of several highly specialized subtypes of macrophages that perform unique tissue-specific functions^1^. Splenic red pulp macrophages, liver Kupffer cells, microglia, and alveolar macrophages are prototypical examples of these specialized tissue-resident macrophage populations. The lungs, which perform gas exchange while being continuously exposed to inhaled pathogens and debris, are host to one such unique population of phagocytes termed alveolar macrophages (AM). AM are positioned on the luminal surface of the alveolar epithelium, where they are uniquely adapted as front-line, sentinel immune cells, aiding debris clearance and surfactant metabolism^2^. Originating from fetal liver monocytes, these precursors seed the embryonic lung and rapidly develop into mature AM post-partum through a burst of proliferation and differentiation that translates into a full complement of AM being present in the lung as early as postnatal day 3 in mice^3,4^. These AM completely self-renew with very little input from peripheral blood monocytes under steady-state conditions^3,5,6^. However, self-renewing tissue-resident AM eventually give way to monocyte-derived AM as they lose their ability to replenish their numbers due to cell senescence^3,6,7^.

In immune defense, AM have been highlighted by studies showing their importance in helping clear pneumococcal infections and controlling inflammation in the lung, and aiding in post-infection recovery^8–10^. AM also metabolizes pulmonary surfactant that is constantly secreted by type-II pneumocytes in the alveolar epithelium^4^. Pulmonary surfactant is primarily composed of phosphatidylcholine, dipalmitoyl phosphatidylcholine, phosphatidylglycerol, various other free fatty acids, and surfactant proteins^11^. This composition of pulmonary surfactant functions to ensure efficient gas exchange and lower surface tension to prevent atelectasis, during expiration^12^. While most of this surfactant is taken up and recycled by type-II pneumocytes, about 20% of it is taken up by AM and broken down into cholesterol and other by-products, which are then expelled into the alveoli^13^. Pulmonary alveolar proteinosis (PAP) is a surfactant clearance disorder that is caused by an inability or lack of AM to process surfactant, resulting in protein and mucous accumulations in the alveolar space, which without regular intervention eventually leads to death due to gas exchange failure^14^. The importance of AM in PAP and the processing of surfactant was highlighted in studies conducted with granulocyte–monocyte colony-stimulating factor-receptor deficient (*Gmcsfr1*^*−/−*^) mice, which manifest classical PAP symptoms, due to a failure in the development of AM, resulting in a very small and dysfunctional population of foam-like AM that are unable to metabolize pulmonary surfactant^3,4^.

Peroxisome proliferator-activated receptor-γ (PPAR-γ) induction through constant GM-CSF signaling is necessary for the proper development and inherent metabolic function of AM required for surfactant metabolism^15^. Other factors such as PU.1^4^, BACH2^16^, BHLEH40/41^17^, mTORC^18^, CEBPβ^19^, and autocrine secretion of TGFβ^20^ are critical for proper development, self-renewal, and imprinting of unique metabolic signatures of AM. Experiments from various gene-deficient mice showing the absence of GM-CSF signaling or signaling of the above-mentioned factors result in the formation of foam-like AM with the drastically impaired development and metabolic function that is characterized by either low numbers of AM or their complete absence^3,4,15^. Recent reports have highlighted the complex interplay between secreted cytokines, cell–cell interaction, and immune-epithelial communication, all of which can contribute to the acquisition of AM-specific identity^21–23^. This includes receptor–ligand interactions between AM and cells lining the alveoli. However, the identification of all such surface receptors on AM is incomplete.

The mouse NKR-P1 receptor family comprises the activating receptors, NKR-P1-A, -C, and -F, which stimulate NK cell cytotoxic immune responses^24–26^, and NKR-P1-B/D (alleles), and -G, which inhibit NK cell responses^27–29^. The NKR-P1 receptors are inherited together with their ligands Clr-b, -c, -d, -f, and -g as part of the NK gene complex on mouse chromosome 6, and exhibit known interactions between NKR-P1B/D:Clr-b; NKR-P1F:Clr-c,d,g; and NKR-P1G:Clr-d,f,g^30,31^. Clr-b engagement by the ITIM-containing NKR-P1B/D receptor results in the recruitment of the phosphatase, SHP-1, which transmits downstream signaling to inhibit NK cell function^32,33^. The NKR-P1B/D:Clr-b interaction acts as an MHC I-independent recognition system that protects potential target cells from NK cell-mediated cytotoxic responses, as well as a regulatory mechanism to protect against immune hyper-reactivity during immune surveillance^29,34,35^. While the function and distribution of these receptor families are best described on classical NK cells, evidence shows that certain ‘NK cell receptors’ are present on other immune cell types and perform critical regulatory functions, such as the expression of NKR-P1C (NKT cells and ILC)^36^, and Ly49Q (on plasmacytoid dendritic cells and neutrophils)^37–40^.

In this study, we demonstrate that resident AM express the NK cell-associated receptor NKR-P1B, and using *Nkrp1b*^*−/−*^ mice, we show that the loss of this receptor has significant functional consequences on the development and metabolism of AM. NKR-P1B loss leads to increased susceptibility to *S. pneumoniae* infection due to a gradual collapse of tissue-resident AM, followed by a period of peripheral blood monocyte-mediated renewal. The collapse of the population of AM is followed by an accumulation of foam-like AM in the alveolar spaces of NKR-P1B-deficient mice that can be resolved by removing them from the lipid-rich alveolar environment. Similarly, *Nkrp1b*^*−/−*^ AM take up exogenous lipid at a higher rate, while also experiencing metabolic and cell-cycle dysregulation, highlighting the importance of NKR-P1B signaling on the lipid processing ability of lung resident AM. We also identify Clr-g expressed on lung epithelial cells (type-II pneumocytes) as a potential ligand for NKR-P1B, whose interaction with NKR-P1B represents a further tissue-specific regulation mechanism that imprints AM with their unique metabolic signature and signaling. Our findings demonstrate tissue-specific signaling through classical ‘NK cell receptors’ that are required for the immune reactivity and homeostatic maintenance of AM. These findings identify potential therapeutic targets and further progress toward understanding how tissue-specific ligands are required for immunosurveillance and immune metabolism.

## Results

### NKR-P1B-deficient mice exhibit significant mortality and morbidity upon *S. pneumoniae* infection

**Fig. 1:**
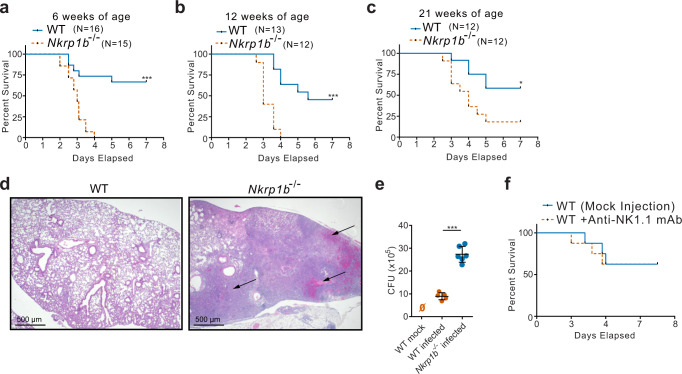
NKR-P1B-deficient mice exhibit greater morbidity and mortality upon pneumococcal infection. **a**–**c** Kaplan–Meyer curves of WT versus *Nkrp1b*^*−/−*^ mice susceptibility to *S. pneumoniae* at 6-, 12-, and 21 weeks of age, respectively. WT (*n* = 16, 13, and 12, respectively), *Nkrp1b*^−/−^ (*n* = 15, 12, and 12, respectively). *p ≤ 0.005, ***p ≤ 0.001. **d** Images of mouse lungs stained with H/E, 3 days post-*S*. *pneumoniae* infection. Images are representative of three separate experiments. Arrows indicate areas of severe alveolar inflammation, bronchopneumonia, and obstructive changes compounded with the destruction of local vasculature causing bleeding to the alveolar space. 500 μm scale bars shown. **e** CFU counts of WT and *Nkrp1b*^*−/−*^ mice 3 days post pneumococcal infections as determined by plating lavage fluid on blood agar plates. Data presented with mean ± SEM. Statistics represent an unpaired, two-tailed Student’s *t*-test. ***p < 0.001 (*n* = 5) for WT and (*n* = 6) for *Nkrp1b*^*−/−*^ mice. **f** Kaplan–Meyer curves of WT mice susceptibility to *S. pneumoniae* after depletion of NK cells via anti-NK.1.1 mAb or a mock injection. (*n* = 8 per group). Source data are provided as a Source Data file.

NKR-P1B is expressed broadly on immune cells and is involved in anti-bacterial responses in the gut and potentially in the lung as well^35,40–42^. Based on these observations, we investigated the requirement for NKR-P1B in respiratory immune responses by infecting 6-week-old WT and *Nkrp1b*^*−/−*^ littermates with *Streptococcus pneumoniae*. While ~70% of WT mice were able to clear and survive *S. pneumoniae* infection, *Nkrp1b*^*−/−*^ mice reached humane endpoints by day 4 post-infection resulting in a 100% fatality rate (Fig. 1a). An inability to clear the pneumococcal infection was also observed in 12-week-old *Nkrp1b*^*−/−*^ mice infected with *S. pneumoniae*, with a 24-h delay until the onset of mortality (Fig. 1b). A slight increase in mortality was observed in 12-week-old WT mice upon infection but it is not uncommon to see dysfunctional immune responses in aged mice^43,44^. *Nkrp1b*^*−/−*^ mice infected at 21 weeks of age show a decrease in mortality compared to infections at earlier ages, yet still showed significantly higher mortality than observed in 21-week-old WT mice (Fig. 1c). To assess the cause of this enhanced mortality, we examined gross lung pathology of *Nkrp1b*^*−/−*^ mice post-infection. *Nkrp1b*^*−/−*^ mice showed significant alveolar epithelial damage as seen in Fig. 1d compared to WT. Pathological scoring and analysis of lung sections from infected 6 and 12-week-old WT and *Nkrp1b*^*−/−*^ littermates also showed an increase in inflammation in *Nkrp1b*^*−/−*^ mice (Supplementary Fig. 1a–d). To extrapolate on the pathological findings, we assessed the bacterial burden present in the lungs of WT and *Nkrp1b*^*−/−*^ mice 3 days post-infection and we found a significant increase in the bacterial load in lavage fluid of *Nkrp1b*^*−/−*^ mice as seen by a three-fold increase in colony-forming unit (CFU) counts shown in Fig. 1e with WT mice showing similar CFU numbers as previously reported with *S. pneumoniae* infection^44^. As NKR-P1B is expressed on NK cells, we assessed whether this reduced survival upon bacterial challenge was NK cell-related. WT mice infected with *S. pneumoniae* after NK cell depletion (using NK1.1 mAb, PK136) showed that there was no difference in survival compared to mock-depleted WT mice, thus excluding NKR-P1B deficiency on NK cells as a source of this observed survival disparity (Fig. 1f). The above data indicate that mice deficient in NKR-P1B exhibit dramatic loss of ability to resist pneumococcal infection and increased bacterial burden resulting in significantly elevated mortality, independent of classical NK and NKT cells.

### Alveolar macrophages express NKR-P1B and NKR-P1B deficiency results in a gradual loss of AM followed by a CCR2-dependent recovery

**Fig. 2:**
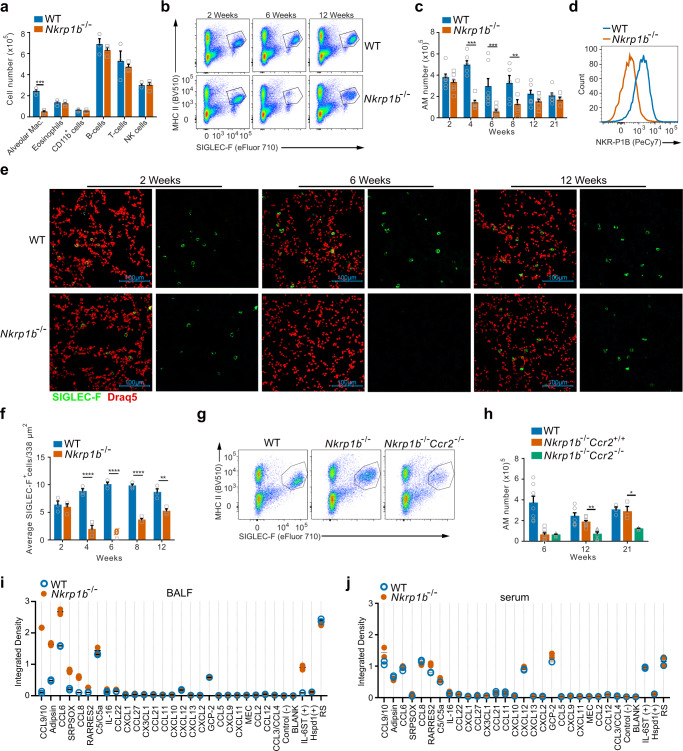
AM express NKR-P1B and NKR-P1B-deficient mice show a gradual loss of AM followed by a CCR2-dependent recovery. **a**Number of various immune cells present in the lungs of WT and *Nkrp1b*^−/−^ mice at 6 weeks of age as determined by flow cytometric phenotyping. Data presented as mean ± SEM. Significance determined by unpaired, two-tailed Student’s *t*-test. ***p < 0.001 (*n* = 3 biologically independent samples). **b** Flow cytometry dot plots of the numbers of AM from WT and *Nkrp1b*^*−/−*^ mice at 2, 6, and 12 weeks of age. Images representative of 7 different experiments. **c** Quantifi**c**ations of AM obtained by flow cytometric analysis from WT and *Nkrp1b*^*−/−*^ mice at 2, 4, 6, 8, 12, and 21 weeks of age (*n* = 7 mice per age per genotype). Data presented as mean ± SEM. Statistical significance was determined by two-way ANOVA with Tukey’s correction where *p* = 0.0011, **p < 0.005, and ***p < 0.0005. **d** Histogram of NKR-P1B surface expression on WT and *Nkrp1b*^*−/−*^ AM. **e** Compiled stack images of WT and *Nkrp1b*^*−/−*^ lungs stained with anti-SIGLEC-F antibody (green) and the nuclear stain DRAQ5 (Red). 100 μm scale bars shown. Images are representative of 6 experiments. **f** Quantifications of AM present in the lungs of WT and *Nkrp1b*^*−/−*^ mice as determined by confocal imaging of immunofluorescent stains in (**e**) (*n* = 6 mice per age per genotype). Data presented as mean ± SEM. Data presented as a number of SIGLEC-F positive cells/338 µm^2^. Statistics were determined by two-way ANOVA with Tukey’s correction where *p* = 0.0094, ***p < 0.001, and ****p < 0.0001. **g** Flow cytometry plots of AM found in the lungs of 12-week-old WT, *Nkrp1b*^*−/−*^, and *Nkrp1b*^*−/−*^*Ccr2*^*−/−*^ mice. Images representative of five experiments. **h** Quantification of AM from 12-week-old WT, *Nkrp1b*^*−/−*^ and *Nkrp1b*^*−/−*^*Ccr2*^*−/−*^ mice. Data presented as mean ± SEM. *n* = 7. **i** and **j** Quantification of chemokine dot blot array performed on WT and *Nkrp1b*^*−/−*^ lavage fluid (BALF) or serum derived from 6-week-old mice. In all graphs and histograms, WT is blue, *Nkrp1b*^*−/−*^ is orange, and *Nkrp1b*^*−/−*^*Ccr2*^*−/−*^ is green. Source data are provided as a Source Data file.

The NKR-P1B requirement for clearance of *S. pneumonia* led us to explore whether any constituents of the lung immune milieu were detectably altered in *Nkrp1b*^−*/*−^ mice at a steady state. This analysis showed a surprising decrease in the number of alveolar macrophages (AM) in *Nkrp1b*^*−/−*^ mice, while levels of other major immune cells remained unchanged (Fig. 2a). Resident AM are at the frontline of pulmonary defense against bacterial pathogens and therefore develop very early in mice, becoming terminally differentiated and present in large numbers by 2 weeks of age^3,5^. Thus, we next determined how NKR-P1B loss affects the population kinetics of AM relative to the age of the mice. As seen in Fig. 2b, AM (gated on live CD45^+^, SIGLEC-F^+^, MHC II^lo^, see Supplementary Fig. 10 for full gating strategy) in *Nkrp1b*^*−/−*^ mice show a dramatic reduction at 6 weeks of age. Quantifications of flow cytometry results show approximately 400,000 AM in the lungs at 2 weeks of age, which then stabilizes to around 250,000 cells/lung up until 21 weeks in WT mice. Conversely, the number of AM in *Nkrp1b*^*−/−*^ mouse lungs at 2 weeks of age are similar to WT numbers, but decline significantly with age, with the lowest number of AM detected at 6 weeks of age before the lung is re-populated at 12 weeks (Fig. 2c). In agreement with these observations, anti-SIGLEC-F immunofluorescent staining of mouse lungs showed AM (large, SIGLEC-F^+^ cells) distributed throughout the lungs of WT mice at all ages, while *Nkrp1b*^*−/−*^ mouse lungs showed a significant decrease in detectable AM at 6 weeks of age followed by subsequent reconstitution (Fig. 2e and f). Conversely, we identified a proportional increase in interstitial macrophages in *Nkrp1b*^*−/−*^ mouse lungs (Supplementary Fig. 2a and b). To examine whether NKR-P1B expression on macrophages was directly associated with the observed changes in *Nkrp1b*^*−/−*^ lung macrophage numbers, we assessed the expression of NKR-P1B on macrophages (using 2D12 mAb). We detected NKR-P1B on resident AM (Fig. 2d), but not on lung interstitial macrophages (Supplementary Fig. 2c), nor on Kupffer cells, splenic red pulp macrophages, or on either large or small peritoneal macrophages, which also did not show any change in numbers in *Nkrp1b*^*−/−*^ mice at 6 weeks of age (Supplementary Fig. 2d–k). Interestingly, we did not find NKR-P1B expression on either Ly6C^+^ or Ly6C^−^ populations of circulating monocytes (Supplementary Fig. 2l and m) either. We also noticed a corresponding age-associated change in the phenotype of AM in *Nkrp1b*^*−/−*^ mice, characterized by a sharp increase in CD11b expression, and a small decrease in F4/80 expression (Supplementary Fig. 2n). These findings demonstrate that NKR-P1B expression is exclusive to lung-resident, fetal-liver monocyte-derived AM and is crucial for their homeostasis.

Resident AM are known to be self-renewing under steady-state conditions, with little input from blood monocytes^3,4,6^. This mechanism is GM-CSF-driven and thus we examined the GM-CSF content in alveolar lavage fluid from 2-week and 6-week-old *Nkrp1b*^*−/−*^ mice, as well as the levels of GM-CSF receptor on *Nkrp1b*^*−/−*^ AM. There were no significant differences observed in lavage fluid GM-CSF content nor GM-CSF receptor expression between WT and *Nkrp1b*^*−/−*^ mice (Supplementary Fig. 2o and p). To assess if GM-CSF/JAK/STAT signaling was affected in *Nkrp1b*^*−/−*^ mice, we measured phosphorylated STAT5 (pSTAT5) levels in AM. However, no significant difference in the level of pSTAT5 of WT and *Nkrp1b*^*−/−*^ AM were detected (Supplementary Fig. 2r), suggesting that the observed decline in the number of AM in *Nkrp1b*^−*/*−^was not due to a breakdown in GM-CSF-mediated self-renewal.

Under certain circumstances, such as lower respiratory tract infections, blood monocytes infiltrate the alveolar niche, through a CCR2-mediated mechanism and differentiate into fully functioning AM^45,46^. To assess the origin of the reconstituted macrophage population, we generated CCR2/NKR-P1B double knockout mice (*Nkrp1b*^−*/*−^*Ccr2*^−*/*−^). The lungs of WT, *Nkrp1b*^−*/*−^*Ccr2*^*+/+*^and *Nkrp1b*^−*/*−^*Ccr2*^−*/*−^ littermates were analyzed by flow cytometry. AM have previously been shown to not express CCR2 and do not depend on it for migration, however, peripheral blood monocytes do^47^. At 12 weeks, the number of AM  in the lungs of *Nkrp1b*^−*/*−^*Ccr2*^*+/+*^ mice were similar to WT numbers, while the number of AM in the lungs of *Nkrp1b*^−*/*−^*Ccr2*^−*/*−^ mice remained significantly depleted at 6 weeks and later, with only marginal increases at 21 weeks of age (Fig. 2g and h). Together, these data show that AM not only express NKR-P1B, but that loss of NKR-P1B leads to a progressive decrease in the number of resident AM beginning at approximately 4 weeks of age, followed by a CCR2-dependent reconstitution of the alveolar niche by blood monocytes beginning at 8 weeks of age. We directly assessed the signals prompting recruitment and replenishment of AM by measuring chemokines present in lavage fluid and serum of 6-week-old WT and *Nkrp1b*^−*/*−^ mice (Fig. 2i and j, and Supplementary Fig. 3a). We detected an increased presence of CCL9/10, Adipsin (CFD), CCL6, SRPSOX (CXCL16), and CCL8 in lavage fluid of *Nkrp1b*^−*/*−^ mice compared to WT mice at 6 weeks of age, but little difference in serum chemokine profiles. Together, these results imply that NKR-P1B expression is critical for the homeostasis of liver-monocyte-derived tissue-resident AM and that even incoming monocytes which repopulate the alveolar niche are still subjected to homeostatic irregularities.

### NKR-P1B-deficient AM accumulate lipid inclusions that resolve over time in vivo

**Fig. 3:**
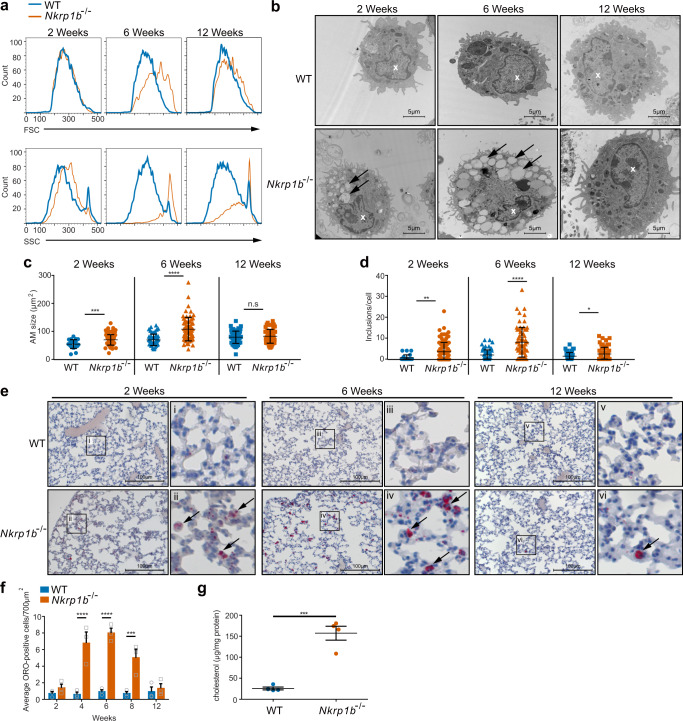
*Nkrp1b*^−*/*−^ AM accumulate intracellular lipids over time. **a** FSC and SSC profiles of AM isolated from WT and *Nkrp1b*^−*/*−^ mice at 2, 6, and 12 weeks of age. **b** Images of AM as seen by TEM isolated from WT and *Nkrp1b*^−*/*−^ mice at 2, 6, and 12 weeks of age. Images are representative of three independent experiments. Inclusions are highlighted by arrows; the nucleus is highlighted with a white “x”. **c** and **d** Quantifications of AM surface area and a number of electron-poor inclusions, respectively, as seen by TEM. AM isolated from mice aged 2, 6, and 12 weeks. Data presented as mean ± SEM. Statistics in **c** and **d** were determined by an unpaired, two-tailed Student’s *t*-test where *p < 0.05, **p < 0.01, ***p < 0.001, and ****p < 0.0001. *n* = 3 with 55 AM examined per experiment. **e** Lung sections stained with ORO obtained from WT and *Nkrp1b*^−*/*−^ mice 2, 6, and 12 weeks of age at ×20 magnification. Boxed areas (i–vi) within each image are shown in magnified panels to the right. Arrows indicate ORO-positive staining. Images are representative of three independent experiments. **f** Quantifications of ORO-positive cells/700 μm^2^ in frozen lung sections stained with ORO obtained from WT and *Nkrp1b*^−*/*−^ mice at 2, 4, 6, 8, and 12 weeks of age. For WT and *Nkrp1b*^−*/*−^ *n* = 3 mice per age. Data presented as mean ± SEM. **g** Cholesterol content of WT and *Nkrp1b*^−*/*−^ lavaged AM as determined by amplex-red assays. Data presented as mean ± SEM. *n* = 4 WT and *Nkrp1b*^−*/*−^ mice. Statistics were determined by an unpaired, two-tailed Student’s *t*-test where ***p < 0.001. All graphs and histograms  indicate WT in blue and *Nkrp1b*^−*/*−^ in orange. Source data are provided as a Source Data file.

As part of the flow cytometric analysis on primary AM from WT and *Nkrp1b*^−*/*−^ mice, we assessed AM forward scatter (FSC) and side scatter (SSC) characteristics. These measurements show a trend of increasing cell size and complexity in AM, peaking in 6-week-old *Nkrp1b*^−*/*−^ mice relative to their WT counterparts (Fig. 3a). To better examine these changes in *Nkrp1b*^−*/*−^ AM morphology, we performed transmission electron microscopy (TEM) analysis of lavaged AM. TEM images show that *Nkrp1b*^−*/*−^ AM contains many electron-poor inclusions in the cytoplasm at 6 weeks compared to WT AM, with some inclusions also present at both 2- and 12-week time points (Fig. 3b). Quantification of AM inclusions and cell size shows that AM is larger and more complex in *Nkrp1b*^−*/*−^ mice at all ages compared to WT mice (Fig. 3c, d), corroborating our cursory FSC/SSC data from Fig. 3a.

A well-known secondary function of AM is processing pulmonary surfactant^13,48^. Considering the electron-poor nature of their inclusions, we postulated that *Nkrp1b*^−*/*−^ AM were accumulating pulmonary surfactant. To this end, we stained frozen lung sections of WT and *Nkrp1b*^−*/*−^ mice with the neutral lipid stain, oil-red-O (ORO). AM from *Nkrp1b*^−*/*−^ mice exhibited many ORO-positive intracellular accumulations compared to WT mice (Fig. 3e), which appear at around 4 weeks of age, peak in number at 6 weeks of age, and decline at 12 weeks of age (Fig. 3f). We further characterized the nature of the accumulated intracellular lipids present in AM from WT and *Nkrp1b*^−*/*−^ mice at 6 weeks of age, by staining lavaged AM with amplex-red, using a protocol designed specifically to measure free cholesterol content. We found that *Nkrp1b*^−*/*−^ AM exhibited a significant increase in the level of intracellular free cholesterol compared to WT AM (Fig. 3g). CD36 is expressed by AM and is known to be a lipid scavenging receptor and a major determinant in lipid uptake kinetics of AM^49,50^. Thus, we examined the level of CD36 on *Nkrp1b*^−*/*−^ AM and found a slight increase in CD36 expression compared to WT AM (Supplementary Fig. 3b), suggesting a possible mechanism for the increased lipid content of AM. Taken together, the above data indicate that resident *Nkrp1b*^−*/*−^ AM accumulate free cholesterol and perhaps other lipids that are present in, or derived from, pulmonary surfactant. As a result, AM of *Nkrp1b*^−*/*−^ mice acquire a foam cell phenotype, and the observed increase in cell size and lipid accumulation aligns with the decline in their population numbers, a characteristic resemblant of cell death caused by lipo-toxicity.

### NKR-P1B-deficient AM experience cell cycle disruption that impairs their ability to self-renew in vivo while showing normal proliferative capacity in vitro

**Fig. 4:**
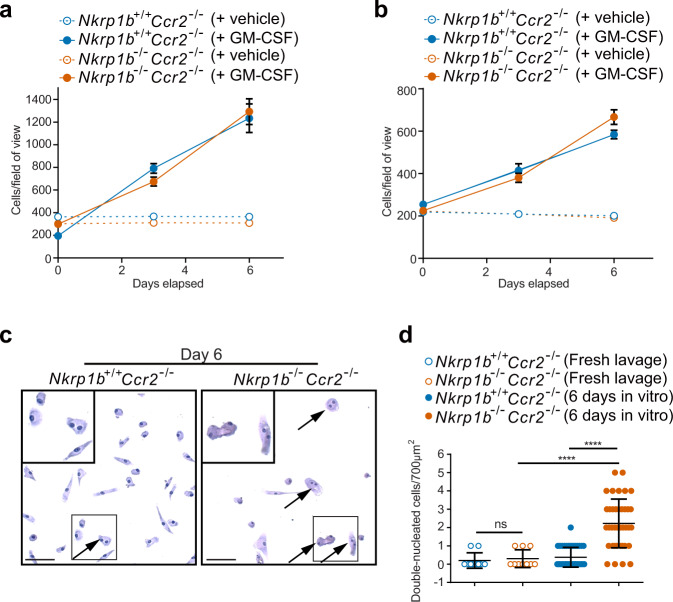
*Nkrp1b*^−*/*−^ AM show signs of cell cycle deficiency in vivo, but not in vitro. **a** and **b** Quantification of a number of visible cells per field of view taken from *Nkrp1b*^+/+^*Ccr2*^−/−^ and *Nkrp1b*^−*/*−^*Ccr2*^−*/*−^ mice at 2 and 6 weeks of age, respectively (*n* = 4 mice per treatment group of each age). Cell numbers were quantified on days 0, 3, and 6 post-plating. Data presented as mean ± SEM. **c** Images of AM isolated from *Nkrp1b*^+/+^*Ccr2*^−/−^ and *Nkrp1b*^−*/*−^*Ccr2*^−*/*−^ mice after being kept in vitro for 6 days and stained with hematoxylin. *Nkrp1b*^−*/*−^*Ccr2*^−*/*−^ frame highlighting double nucleated cells (arrows). Select cells within inset boxes are shown magnified in the top left corner. Images are representative of three independent experiments. 50 μm scale bars are shown. **d** Quantifications of double-nucleated cells as seen in **c** from freshly lavaged AM from *Nkrp1b*^+/+^*Ccr2*^−*/*−^ and *Nkrp1b*^−*/*−^*Ccr2*^−*/*−^ (*n* = 2 mice per genotype) or AM lavaged from *Nkrp1b*^+/+^*Ccr2*^−*/*−^ and *Nkrp1b*^−*/*−^*Ccr2*^−*/*−^ and kept in vitro for 6 days (*n* = 4 mice per genotype). Data presented as mean ± SEM. Statistics were determined by an unpaired, two-tailed Student’s *t*-test where ****p < 0.0001. In all graphs, *Nkrp1b*^+/+^ is indicated in blue and *Nkrp1b*^−*/*−^ in orange. Source data are provided as a Source Data file.

Since *Nkrp1b*^−*/*−^ AM accumulate surfactant, which has the potential to cause lipo-toxicity, we decided to investigate whether AM isolated from the lungs could be induced to proliferate if removed from the lipid-rich alveolar environment. Visual quantification of lavaged AM as seen in Supplementary Fig. 3c showed no proliferation in vitro when GM-CSF is absent from the growth medium, but upon the addition of GM-CSF, AM of 2-week-old *Nkrp1b*^*+/+*^*Ccr2*^−*/*−^ and *Nkrp1b*^−*/*−^*Ccr2*^−*/*−^ mice showed a similar proliferation rate over the entire 6-day period (Fig. 4a). This was also true for AM extracted from 6-week-old mice (Fig. 4b). Interestingly, while both *Nkrp1b*^*+/+*^*Ccr2*^−*/*−^ and *Nkrp1b*^−*/*−^*Ccr2*^−*/*−^ lavaged AM were able to survive ex vivo, *Nkrp1b*^−*/*−^*Ccr2*^−*/*−^ AM exhibited double nuclei at a frequency that was not observed in WT AM after 6 days of in vitro culture without GM-CSF (Fig. 4c and d) suggesting an inherent cell cycling defect in *Nkrp1b*^−*/*−^*Ccr2*^−*/*−^ AM. Flow cytometric BrdU analysis of lavaged AM from *Nkrp1b*^−*/*−^*Ccr2*^−*/*−^ mice shows an accumulation of AM in the S-phase of cell division, with *Nkrp1b*^−*/*−^*Ccr2*^−*/*−^ mice having almost 20% of cells lingering in S-phase compared to less than 10% for *Nkrp1*^*+/+*^*Ccr2*^−*/*−^ AM (Supplementary Fig. 3d). As the replenishment of AM in *Nkrp1b*^−*/*−^ mice are mostly CCR2-dependent, we proceeded to assess proliferative capacity using *Nkrp1b*^−*/*−^*Ccr2*^−*/*−^ mice to exclude any possible monocyte infiltration. At 2 weeks of age *Nkrp1b*^−*/*−^*Ccr2*^−*/*−^ AM exhibited lower levels of Ki67, as determined by intracellular flow cytometry than AM of *Nkrp1*^*+/+*^*Ccr2*^−*/*−^ mice (Supplementary Fig. 3e). The above data indicate the presence of a defect in cell cycle or cytokinesis in *Nkrp1b*^−*/*−^ AM which is resolved when the AM are removed from the lipid-rich alveolar environment and forced to proliferate in vitro.

### RNA-Seq and lipidomic analyses indicate NKR-P1B requirement for metabolic processes that protect AM against the accumulation of toxic cellular lipid species

**Fig. 5:**
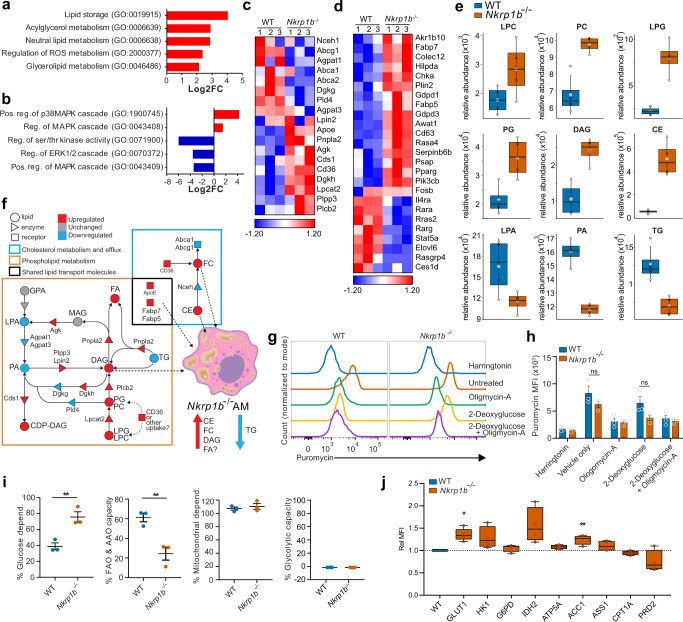
RNA-Seq and lipidomics indicate a dysregulation of metabolism in *Nkrp1b*^−*/*−^ AM. **a** and **b** Gene ontology pathways enriched (Log2 fold change) in *Nkrp1b*^−*/*−^ AM from 2-week-old mice as determined by RNA-Seq analysis. **c** and **d** Heatmaps of select lipid metabolic and general metabolic genes in AM. Log2 fold change enrichment presented as *Z*-scores (*n* = 3 replicates) for WT and *Nkrp1b*^−*/*−^ with a pool of five mice for every replicate. **e** Comparison between the abundance of an indicated lipid species and internal lipid standards from lipidomic analysis of AM from 6-week-old WT and *Nkrp1b*^−*/*−^ mice (*n* = 4 independent experiments per genotype). LPC (Lysophosphatidylcholine), PC (Phosphatidylcholine), LPG (Lysophosphatidylglycerol), PG (Phosphatidylglycerol), DAG (Diacylglyceride), CE (Cholesterol ester), LPA (Lysophosphatidic acid), PA (Phosphatidic acid), TG (Triglyceride). Boxplots indicate median, IQR, maximum, and minimum bars. Large and small data points represent average SD and individual experimental replicates, respectively. **f** Model combining RNA-seq (**c**) and lipidomics (**e**) data showing increased (red) or decreased (blue) lipid species and enzyme/receptor expression in *Nkrp1b*^−*/*−^ AM. Dashed arrows indicate lipid shuttling into droplets. **g** Representative histograms of puromycin incorporation into WT and *Nkrp1b*^−*/*−^ AM from 2-week-old mice exposed to Harringtonin, Vehicle/untreated control, Oligomycin-A, 2-Deoxyglucose, 2-Deoxyglucose + Oligomycin-A (*n* = 3 independent experiments). **h** MFI quantification of puromycin incorporation into AM as shown in (**g**). Data presented as mean ± SEM, each data point represents an independent experiment of 3 mice of each genotype pooled. Statistics represent two-way ANOVA with Sidak’s correction (*n* = 3). **i** Percent glucose dependence and FAO/AAO capacity in WT and *Nkrp1b*^−*/*−^ AM derived from MFIs in (**h**) (*n* = 3 independent experiments). See methods for equations. Data presented as mean ± SEM. Statistics in **i** represent unpaired, two-tailed Student’s *t*-test; *p < 0.05 and **p < 0.01. **j** Relative MFI of stained metabolic proteins in WT and *Nkrp1b*^−*/*−^ AM (*n* = 4 mice per genotype per stain). Boxplots indicate mean, IQR, and data minimum and maximum whiskers. Statistics represent unpaired, two-tailed Student’s *t*-test of data prior *t*o normalization; *p < 0.05 and **p < 0.01. Source data are provided as a Source Data file.

Two obvious defects in *Nkrp1b*^−*/*−^ AM are apparent: lipid processing and cell cycle. To identify the cellular programs that underlie the observed defects of *Nkrp1b*^−*/*−^ AM, we performed RNA-Seq analysis on AM sorted from 2-week-old WT and *Nkrp1b*^−*/*−^ mouse lungs. Altogether, 590 genes were significantly dysregulated (Supplementary Fig. 4a). Gene ontology analysis on this data set show a pattern of dysregulation of gene expression characterized by excessive lipid storage and upregulation of various lipid metabolic pathways (Fig. 5a and Supplementary Fig. 4b). As well, kinase activity was found to be dysregulated, as indicated by an apparent increase in factors regulating positive p38-MAPK loops, and a decrease in the ERK cascade and the factors regulating it (Fig. 5b and Supplementary Fig. 4c). The transcriptional profile of *Nkrp1b*^−*/*−^ AM showed altered expression of genes that encode well-characterized components of lipid processing and efflux, such as ABCA1, ABCG1, and DAG-kinases, as well as lipases such as LPCAT2 and PNPLA2 (Fig. 5c). Other dysregulated genes such as *Cd36, Lpin2, Apoe*, and *Cds1* mediate the uptake and conversion of lipids from one species into another (Fig. 5c). We noted the downregulation of *Stat5a*, *Rara*, *Rarg*, and *Il4ra* and the upregulation of *Ppar-γ* (Fig. 5d). These genes have been shown to have significant consequences on the metabolic programming and differentiation of AM^15,16,51^. Further, we identified an upregulation in the expression of mediators of lipid metabolism in AM. In particular, *Akrb10, Hilpda, Plin2, Fabp5*, and *Serpinb6b*, were upregulated in *Nkrp1b*^−*/*−^ AM, all of which are involved in lipid transition and metabolism, as well as lipid droplet formation. Finally, our RNA-Seq analysis showed significant dysregulation in cell cycling with many critical cell cycle pathways downregulated (Supplementary Fig. 4d and 4e), in agreement with our flow cytometry findings.

In support of the RNA-Seq analysis, lipidomic analysis of 6-week-old *Nkrp1b*^−*/*−^ AM showed dysregulation in several critical lipid classes. Levels of lysophosphatidylcholine (LPC), phosphatidylcholine (PC), lysophosphatidylglycerol (LPG), and phosphatidylglycerol (PG) were considerably higher in *Nkrp1b*^−*/*−^ AM (Fig. 5e). Likewise, a significant accumulation of diacylglycerols (DAG) and cholesterol esters (CE) was detected in *Nkrp1b*^−*/*−^ AM, while the amount of lysophosphatidic acid (LPA), phosphatidic acid (PA), and triglycerides (TG) were lower in *Nkrp1b*^−*/*−^ AM. Combining the transcriptomic data with our lipidomic findings allows us to reconstruct and examine changes in relevant metabolic networks that define functions in AM. As illustrated in Fig. 5f, the upregulation of CD36 and perhaps the activity of other receptors as well may account for the increased uptake of PC, PG, cholesterol, and perhaps LPC and LPG, which together comprise the bulk of surfactant lipids. Also, our findings indicate the dysregulation of lipid metabolic processes in *Nkrp1b*^−*/*−^ AM, which is supported by the upregulation of enzymes such as PLCB2, PNPLA2, and PLPP3, which catalyze the breakdown of TG, PA, and LPA by converting them into DAG and various fatty acids, lipid classes that are respectively diminished and increased in our analysis. This shunt towards DAG accumulation in *Nkrp1b*^−*/*−^ AM, combined with a deficiency of DGKG, serves to create an excess of DAG species shuttled into lipid droplets along with the canonical lipid storage species of CE, which is also found in excess in *Nkrp1b*^−*/*−^ AM. However, the low TG lipid pool, considered to be a safe storage lipid, is indicative that lipid droplets in *Nkrp1b*^−*/*−^ AM contain species of DAG, FC, and various fatty acids in excess, which are known to induce lipo-toxicity^52,53^.

The substantial dysregulation in transcriptional programs related to metabolic processes in *Nkrp1b*^−*/*−^ AM and the notable changes in the lipid profile of AM in *Nkrp1b*^−*/*−^ mice points to an underlying dysregulation of cellular metabolic processes. To test this, we performed a flow cytometry-based profiling of energetic metabolism in AM of WT and *Nkrp1b*^−*/*−^ mice using the SCENITH assay^54^. Specifically, AM lavaged from 2-week-old WT and *Nkrp1b*^−*/*−^ mice were treated with either an ATP synthase inhibitor, oligomycin-A, or a competitive inhibitor of glycolysis, 2-deoxyglucose, or a combination of both following incubations with puromycin. Flow cytometry measurement of puromycin incorporation into nascent proteins, a reliable measure of protein synthesis, showed a modest but nonsignificant decrease in protein synthesis only in *Nkrp1b*^−*/*−^ AM treated with 2-deoxyglucose (Fig. 5g and h). However, calculation of the metabolic dependencies and capacities using this data shows a significantly elevated glucose dependency of *Nkrp1b*^−*/*−^ AM, and a significantly decreased fatty acid and amino acid oxidation capacity compared to WT AM. Additionally, both WT and *Nkrp1b*^−*/*−^ AM exhibit a maximal mitochondrial dependency, but minimal glycolytic capacity (Fig. 5i). We further examined the metabolic status of WT and *Nkrp1b*^−*/*−^ AM by measuring rate-limiting enzymes across multiple metabolic pathways using the flow cytometry-based Met-Flow antibody panels^55^. From these assays, we observed a significant increase in the glucose uptake transporter, Glut1, and acetyl-CoA carboxylase alpha (ACC1) the rate-limiting enzyme in de novo long-chain fatty-acid synthesis (Fig. 5j). Together, our metabolic profiling data indicates a shift in the metabolism of *Nkrp1b*^−*/*−^ AM towards elevated glycolysis and possibly lipogenesis, and a decrease in lipid catabolism.

### NKR-P1B signaling regulates critical metabolic genes responsible for lipid turnover

**Fig. 6:**
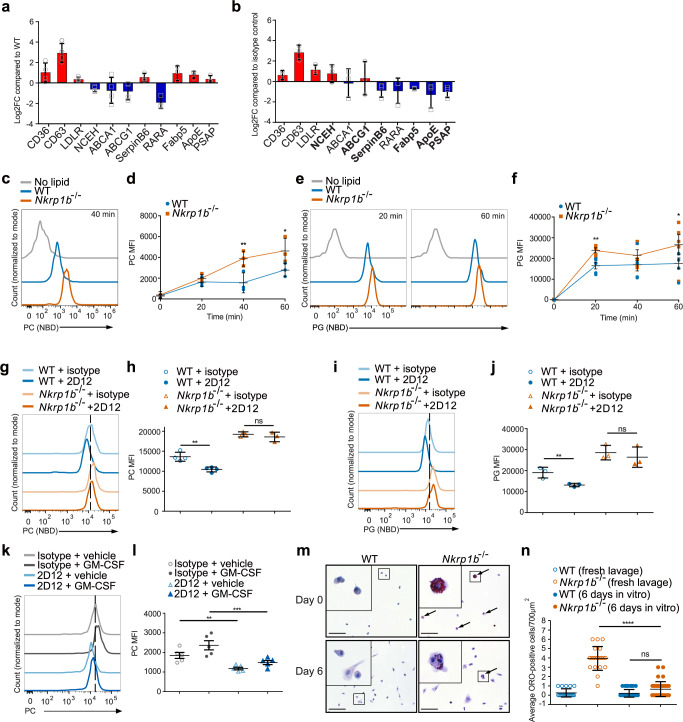
NKR-P1B signaling correlates with the rate of surfactant lipid uptake by AM. **a** RT-qPCR of select metabolic genes from WT and *Nkrp1b*^−*/*−^ AM from 2-week-old mice. Data normalized to WT levels. **b** RT-qPCR analysis of WT AM crosslinked with 2D12 or isotype antibody. Data normalized to isotype control. **c** Fluorescence of WT and *Nkrp1b*^−*/*−^ AM after 40 min NBD-PC incubation. **d** MFI of WT and *Nkrp1b*^−*/*−^ AM after 0, 20, 40, and 60 min NBD-PC incubation. **e** Fluorescence of WT and *Nkrp1b*^−*/*−^ AM after 20 and 60 min incubation with NBD-PG. **f** MFI of WT and *Nkrp1b*^−*/*−^ AM after 0, 20, 40, and 60 min NBD-PG incubation. **g** NBD-PC uptake in WT and *Nkrp1b*^−*/*−^ AM crosslinked with 2D12 or isotype control. **h** MFI of NBD-PC uptake in (**g**). **i** NBD-PG uptake in WT and *Nkrp1b*^−*/*−^ AM crosslinked with 2D12 or isotype control. **j** MFI of NBD-PG uptake in (**i**). **k** NBD-PC uptake in WT AM crosslinked with 2D12 or isotype control in the presence of GM-CSF or vehicle control. **l** MFI of NBD-PC uptake in (**k**). Data in **a**–**d**, **g**–**j** (*n* = 3 independent experiments). Data in **e**, **f**, **k**, **l** (*n* = 5 independent experiments). **m** Images of WT and *Nkrp1b*^−*/*−^ AM after 0 or 6 days of in vitro culture post-ORO staining. 100 μm scale bars shown. ORO-staining indicated (arrows). **n** Quantification of ORO-positive AM/700 µm^2^ shown in (**m**). Data in **m** and **n** represent (*n* = 2; day 0) or (*n* = 3; day 6) independent experiments. Data in **a** and **b** show mean ± SD and **d**, **f**, **h**, **j**, **l**, **n** show mean ± SEM. Statistics for **d** and **f** represent an unpaired, two-tailed Student’s *t*-test of data prior to normalization where *p < 0.05 and **p < 0.01. Statistics for **h**, **j**, **l**, and **n** represent a two-way ANOVA with Tukey’s correction where **p < 0.01, ***p < 0.001, and ****p < 0.0001. All graphs and histograms indicate WT in blue and *Nkrp1b*^−*/*−^ in orange. Source data are provided as a Source Data file.

To verify the transcriptional dysregulation of specific lipid uptake and metabolic mediators, we performed qPCR analysis on AM of 2-week-old WT and *Nkrp1b*^−*/*−^ mice for the lipid uptake receptor, *Cd36*, the cholesterol efflux cassettes, *Abca1*, *Abcg1*, and the transcription factor, *Rara*, all of which have been highlighted by RNA-seq as being dysregulated. The differential expression results for all the genes selected for qPCR analysis, except for *Ldlr*, corresponded with the observed changes identified by RNA-seq (Fig. 6a). We postulated that if a lack of NKR-P1B results in dysregulation in the genes examined in Fig. 6a, cross-linking the receptor using an anti-NKR-P1B antibody (clone 2D12), thereby forcing signaling through its ITIM, should produce the opposite effect in WT AM. Indeed, cross-linking of NKR-P1B on WT AM resulted in increases of *Nceh* and *Abcg1*, as well as decreases in *Serpinb6b, Fabp5, Apoe*, *and Psap*, compared to unstimulated WT AM (Fig. 6b), all of which showed the opposite effect in qPCRs and RNA-seq performed on *Nkrp1b*^−*/*−^ AM, thus indicating a potentially direct modulatory function for NKR-P1B signaling in these genes. Levels of *Cd36, Cd63, Ldlr, Abca1*, and *Rara* were similar to that of NKR-P1B-deficient AM indicating a secondary effect of NKR-P1B loss. We assayed the p38 MAPK phosphorylation status in *Nkrp1b*^−*/*−^ AM, as the p38 MAPK cascade is a known transcriptional inducer of various scavenger receptors and processes associated with lipid foam cell formation. We observed elevated phosphorylation of p38 MAPK in *Nkrp1b*^−*/*−^ AM (Supplementary Fig. 4f). Taken together, the RNA-seq and lipidomic data highlight a fundamental dysregulation of lipid metabolism, which biases NKR-P1B-deficient AM to store toxic lipid species. The immediate modulation of *Nceh*, *Abcg1*, *Serpinb6*, *Fabp5*, *Apoe*, *Psap*, and *Rara* expression after NKR-P1B activation, points to an interaction between the regulation of lipid processing in AM and NKR-P1B.

### NKR-P1B negatively regulates lipid uptake by AM

While previous data points to an apparent defect in lipid metabolism and storage, the modulation of scavenger receptors by NKR-P1B led us to examine whether NKR-P1B is also required for phagocytic process in AM. Lavaged AM from *Nkrp1b*^−*/*−^ mice did not exhibit notable alteration in phagocytic capacity compared to WT AM (Supplementary Fig. 5a and b). We, therefore, measured lipid uptake kinetics in AM of WT and *Nkrp1b*^−*/*−^ mice. Lavage *Nkrp1b*^−*/*−^ AM showed an increase in both PC and PG uptake in vitro compared to their WT counterparts (Fig. 6c and e) when incubated with NBD-labeled PC or PG for 20, 40, and 60 min. Quantifications show a trend of increased PC and PG uptake in *Nkrp1b*^−*/*−^ AM, which is primarily evident at 40- and 60-min time points and 20- and 60-min time points, respectively (Fig. 6d and f). On this basis, we hypothesized that cross-linking (via 2D12 mAb) of the NKR-P1B receptor on WT AM should produce the opposite effect. Indeed, cross-linking of NKR-P1B on AM derived from WT mice prior to co-incubation with PC resulted in significantly decreased PC uptake (Fig. 6g, and h). Decreases in lipid uptake were not observed when cross-linking AM with an isotype control antibody, nor when using AM derived from *Nkrp1b*^−*/*−^ mice (Fig. 6g and h). Likewise, cross-linking of WT AM prior to co-incubation with PG also resulted in decreased lipid uptake (Fig. 6i and j).

The GM-CSF signaling axis is an established regulator of lipid metabolic processes that could affect lipid uptake in AM. However, our findings in Supplementary Fig. 2o, p, and r showed no significant differences observed in GM-CSF content of lavage fluid, nor differences in GM-CSF receptor expression or pSTAT5 levels between WT and *Nkrp1b*^−*/*−^ AM. Nevertheless, we tested whether changes in lipid uptake could be observed in AM with or without NKR-P1B receptor cross-linking both in the presence or absence of GM-CSF (Fig. 6k and l). We found that while lipid uptake does increase globally in the presence of GM-CSF, it does not appear to have any particular effect when the receptor is activated, suggesting the GM-CSF pathway-mediated alteration of lipid metabolism in AM is separate from NKR-P1B.

The engulfed lipids by AM during routine surfactant turnover and clearance of apoptotic debris in the lung, generate ligands for nuclear receptors that modulate lipid metabolism and phagolysosome processing^56^. Given the observed changes in both lipid uptake and expression of transcriptional activators of lipid metabolism in *Nkrp1b*^−*/*−^ AM, we assessed if NKR-P1B altered phagolysosome formation. The lavaged AM from 6-week-old *Nkrp1b*^−*/*−^ mice showed no significant changes in the distribution or number of phagolysosomes in AM (Supplementary Fig. 5c and d).

Given that *Nkrp1b*^−*/*−^ AM show elevated lipid uptake within a lipid-rich environment we assessed if the lipid-laden phenotype of *Nkrp1b*^−*/*−^ AM could be resolved if removed from the lipid-rich alveolar environment. To test this, we cultured lavaged AM in vitro for 6 days, then stained them with ORO and analyzed them by microscopy side-by-side with freshly lavaged AM as a positive ORO control. As can be seen in images in Fig. 6m, WT AM have little to no ORO positivity either when freshly lavaged or after 6 days in vitro. Conversely, *Nkrp1b*^−*/*−^ AM shows a dramatic decrease in ORO-positive cells after 6 days in vitro compared to freshly lavaged AM from *Nkrp1b*^−*/*−^ mice, with no correlation, observed between remaining ORO-positivity and a polyploid phenotype. Quantifying these observations confirmed a clear significant reduction in ORO-positive *Nkrp1b*^−*/*−^ AM following 6 days of in vitro culture (Fig. 6n).

Due to a recent publication showing the potential of statin therapy to alleviate metabolic defects in AM in mice with severe PAP^57^, we decided to apply the same treatment to *Nkrp1b*^−*/*−^ mice. After 2 weeks of pravastatin administration, we collected the lungs of WT and *Nkrp1b*^−*/*−^ mice and stained them with ORO. A significant reduction in ORO-positive AM of statin-treated compared to vehicle-treated *Nkrp1b*^−*/*−^ mice was observed (Supplementary Fig. 6a and b). Since *Nkrp1b*^−*/*−^ AM are rescued from their lipid burden by removing them from the surfactant-rich alveolar environment and culturing them in vitro, while lipid burden of *Nkrp1b*^−*/*−^ AM appeared to be reversed by in vivo statin administration, indicating the loss of *Nkrp1b*^−*/*−^ AM metabolic activity can be modulated with the administration of pharmaceuticals. The observations that several *Nkrp1b*^−*/*−^ AM phenotypes were reversible, led us to examine whether *Nkrp1b*^−*/*−^ AM exhibited any intrinsic changes in metabolism. We found the *Nkrp1b*^−*/*−^ AM had a higher glucose uptake than WT AM (Supplementary Fig. 7a), which supports our finding that AMs have elevated glycolytic metabolism (Fig. 5g–j). Also, analysis of *Nkrp1b*^−*/*−^ oxygen consumption rate by AM showed an elevated rate of extracellular oxygen consumption compared to WT AM (Supplementary Fig. 7b). We also detected a modest decrease in mitochondrial membrane potential by TMRE, and mitochondrial mass by MitoTracker staining (Supplementary Fig. 7c and d). To assess if these differences altered oxidative stress, we measured cellular levels of ROS in *Nkrp1b*^−*/*−^ AM and measured NOS-2 levels (Supplementary Fig. 7e and f) and found decreased ROS and slightly elevated NOS-2 in *Nkrp1b*^−*/*−^ AM. Together, these findings are indicative of an intrinsic change in the cellular respiration, mitochondrial status, and potentially glycolysis of AM in *Nkrp1b*^−*/*−^ mice.

### NKR-P1B tetramers bind to Clr-g-expressing type-II pneumocytes in an infection-sensitive manner

**Fig. 7:**
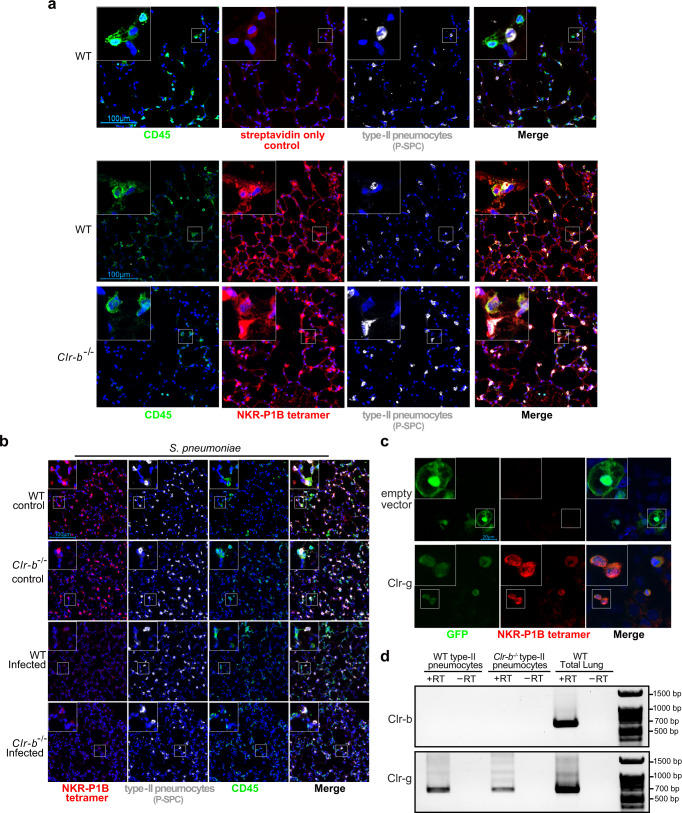
Infection downregulates NKR-P1B ligand expression. **a** Confocal image stacks of WT and *Clr-b*^−*/*−^ frozen lung sections stained with CD45 (Green), NKR-P1B tetramer (Red), DAPI (Blue), and pro-surfactant protein C (White). **b** Confocal image stacks of WT and *Clr-b*^−*/*−^ frozen lung sections post-infection with *S. pneumoniae* harvested 3 days post-infection. Sections are stained with CD45 (Green), NKR-P1B tetramer (Red), DAPI (Blue), and pro-surfactant protein C (White). **c** MLE-12 cells transiently transfected with either empty vector or Clr-g expressing vector. Vector contains a separate GFP message for verification of transfection. Cells were grown on coverslips and stained 48 h post-transfection. **d** RT-PCR amplification of Clr-b and Clr-g transcripts in type-II pneumocytes sorted from WT and *Clr-b*^−*/*−^ lungs. No reverse transcriptase (−RT) controls and WT total lung controls are shown. All data presented are representative of *n* = 3 independent experiments. Source data are provided as a Source Data file.

The only known ligand for NKR-P1B that has been identified to date is Clr-b, but surprisingly, Clr-b deficient mice do not replicate the phenotype observed in *Nkrp1b*^−*/*−^ AM neither in terms of cell numbers nor *S. pneumoniae* challenge at 6-weeks of age (Supplementary Fig. 8a and b). Quantifications of AM isolated from *Clr-b*^−*/*−^ mouse lungs show normal levels of AM present throughout all time points analyzed (Supplementary Fig. 8c). We have also verified that Clr-b deficiency does not disrupt NKR-P1B expression on AM (Supplementary Fig. 8d). The failure of *Clr-b*^−*/*−^ AM to phenocopy *Nkrp1b*^−*/*−^ AM implies that there must be another, lung-specific, interacting partner for NKR-P1B present. Our previous study found high levels of *Clr-g* mRNA present in the lung epithelium^33^ as demonstrated by in situ hybridization and qPCR. The alveolar epithelium is made up of type-I and type-II pneumocytes, and given the intimate relationship between the surfactant-secreting type-II pneumocytes and AM^58–60^, we decided to probe whether these cells express a ligand that our NKR-P1B tetramer could bind to and whether it can serve as a potential interacting partner for NKR-P1B expressed on AM. Staining lung tissue sections of WT and *Clr-b*^−*/*−^ mice with pro-surfactant protein C (P-SPC) antibody (a marker exclusive for type-II pneumocytes) and biotinylated NKR-P1B tetramers (which faithfully stain Clr-b-expressing lymphocytes in mouse tissues as seen in Supplementary Fig. 8e and f), we were able to show co-localization of P-SPC and NKR-P1B tetramer in WT and *Clr-b*^−*/*−^ mouse lungs (Fig. 7a). This is indicative of a type-II pneumocyte-restricted interaction between NKR-P1B and another ligand, with Clr-g being the best candidate based on previous evidence. AM respond to infection with their immune and metabolic programming controlled in part through interactions with pathogens and the alveolar epithelium^61,62^. As such, we wanted to explore whether this potential ligand responds to infection. Confocal imaging of *S. pneumoniae* (Fig. 7b) or influenza A virus-infected (Supplementary Fig. 9) WT and *Clr-b*^−*/*−^ mouse lung sections shows a reduction in NKR-P1B tetramer staining when compared to vehicle controls, thus indicating that type-II pneumocytes respond by downregulating a ligand from the surface of type-II pneumocytes. Finally, to confirm this interaction between AM and type-II pneumocytes, we cultured MLE-12 cells (derived from type-II pneumocytes) on coverslips and transiently transfected them with a construct expressing Clr-g and GFP. Using NKR-P1B tetramers, we were able to confirm the colocalization of tetramers to GFP^+^ MLE-12 cells transfected with the Clr-g expression construct, while no colocalization occurred in the MLE-12 cells transfected with the empty vector (Fig. 7c). To confirm that Clr-g was the specific Clr expressed in type-II pneumocytes, we assayed lung type-II pneumocytes isolated by FACs sorting of WT and *Clr-b*^−*/*−^ lungs and performed RT-PCR analysis for transcript expression of both *Clr-b* and *Clr-g* transcripts, the two Clr family members previously found be expressed in the lung. Our RT-PCR analysis identified *Clr-g* transcript in type-II pneumocytes, while *Clr-b* was absent (Fig. 7d). Together, the above data indicate that type-II pneumocytes express a potential ligand for NKR-P1B on their surface as determined by NKR-P1B tetramer binding and the detection of this ligand on the cell surface of pneumocytes is lost during infection. NKR-P1B tetramers can bind to this ligand, which in all likelihood is Clr-g expressed on type-II pneumocytes, as seen in vivo and recapitulated in vitro, thus establishing a tissue-specific binding partner for NKR-P1B expressed on AM.

## Discussion

The conserved NK cell receptor, NKR-P1B is known to exhibit inhibitory functions on NK cells and to act as a mediator for MHC I-independent NK recognition^28,29^. The discovery that mouse AM rely on NKR-P1B to function and survive, extends both our understanding of this lectin-like receptor and how the alveolar niche supports resident AM. The regulation of resident immune cell activity through the modulation of tissue-specific signals is a hallmark feature of tissue microenvironments. As such, NKR-P1B receptor sensing of dynamically regulated ligands present in alveoli may represent a flexible line of communication between the alveolar epithelium and AM that functions in parallel with other reported AM–epithelial interactions. Previous work has shown that communication between the alveolar epithelium and the lung immune complement is necessary for proper immune functioning. In particular, the interaction between CD200 expressed on AM and CD200R expressed on the alveolar epithelium is necessary for myeloid restraint and aid in AM-mediated inflammation resolution post-infection. Lack of this interaction extended inflammatory processes and increased morbidity and mortality due to run-away inflammation^63^. Similar research conducted by Metzger found that the presence of IFN_Ɣ_ during pulmonary infection has a suppressive effect on both ILC2s^64^ and AM^65^ leading to extended inflammatory processes and poor prognosis during post-IAV bacterial superinfections. Our findings here indicate that the communication between pneumocytes and AM which supports lung immune surveillance, the maintenance of lung AM, and metabolic functions of AM also relies on NKR-P1B receptor expression on AM. The interconnections between innate immune detection and response mechanisms and lipid metabolism are unique attributes of the lung alveolar environment. Surfactant proteins, which are C-type lectin proteins, are intimately involved in both lipid metabolism and lung immunity. Specifically, the surfactant proteins SP-D and SP-A, are able to bind numerous viruses and bacteria, while also having diverse functions in the regulation of innate and adaptive immune responses^66^. These surfactant proteins not only exhibit broad binding capabilities to lipids, carbohydrates, and proteins but also differential immune regulatory functions depending on the level of multimerization. A similar multi-purpose system could have likewise evolved for C-type lectin proteins on AMs. Such a system would permit the ability for pathogen detection, and activation of AM, and permit the induction of a more glycolytic state instead of an anti-inflammatory state without the need for multiple receptors. Notably, the unique function that NKR-P1B performs in resident AM may reflect characteristics that are partly unique to the alveolar niche and partly lineage-specific, as monocyte-derived AM do develop, but do not exhibit synonymous functional attributes observed in the fetal liver-derived resident population.

AM have several well-defined functions in the protection of the lungs from pathogens, such as *S. pneumoniae*, and in the resolution of inflammation following pathogenic insult^8,67,68^. Therefore, taking in mind that *Nkrp1b*^−*/*−^ mice suffer from a collapse in the number of AM, it follows that they also succumb to the pneumococcal challenge. It is important to note that while *Nkrp1b*^−*/*−^ AM reconstitute almost the entire alveolar niche in *Nkrp1b*^−*/*−^ mice they exhibit both phenotypic and immunological defects which may impair their ability to clear *S. pneumoniae* infection despite their number. This finding led us to originally hypothesize that NKR-P1B in AM operates through interaction with Clr-b, the prototypical inhibitory ligand for the NKR-P1B receptor on NK cells. Clr-b expression is downregulated upon infection and cellular stress^27,69,70^, a signal that guides NK cell immune surveillance of peripheral tissues. However, Clr-b does not exhibit a comparable function in the regulation of AM similar to NKR-P1B. While Clr-b transcripts are also present in the lung, their expression has been validated only in lymphocytes. Rather, our data support an alternate binding partner for NKR-P1B in the lung. One potential binding partner, Clr-g, is expressed in type-II pneumocytes and shares similarities with Clr-b^33,34^. Although our evidence here supports Clr-g as an NKR-P1B ligand in the lung, the generation of a Clr-g mutant mouse is needed to establish the definitive nature of this interaction. It is also plausible that a much broader range of receptor/ligand combinations exist between the NKR-P1 and Clr proteins, as the interactions defined to date were largely understood through interaction assays such as BWZ reporter assays, tetramers, and crystallography in overexpression studies^28^, which may not account for optimal post-translational modifications or glycosylation (such as on type-II pneumocytes), which may affect C-type lectin and perhaps lectin-like receptor binding^71–73^. Moreover, the observed downregulation of the NKR-P1B binding partner in the lung during infection has interesting implications for epithelial interactions by AM through NKR-P1B. This downregulation may serve as a signal of epithelial damage instructing AM to shift from surfactant metabolism and homeostasis to an immune active phenotype. Further studies utilizing infection models and cytokine profiling of WT and NKR-P1B-deficient AM could be used to elucidate the specific immunomodulatory impact of NKR-P1B:Clr-g interactions.

The slow progressive decline in the number of AM in *Nkrp1b*^−*/*−^ mice, which occurs over 4–6 weeks, contrasts reports of developmental failures of AM in GM-CSF receptor-, PPAR-γ-, and TGF-β-deficient mice, in which AM are not present in any significant numbers from birth, or they collapse very shortly (7–14 days) post-partum^4,15,20^. Similar findings have also been reported in studies of VHL, mTORC, and BACH2^16,18,74^. Conversely, the knockout of the Bhlhe40/Bhlhe41transcriptional regulators did not alter the number of AM at steady state, despite affecting surfactant metabolism and impairing colonization of the alveolar niche by AM in competitive mixed bone-marrow chimeras^17^. The disruptions in cytokine receptors or transcription factors that do affect the numbers of AM, were largely lethal due to PAP, whereby the residual foamy, lipid-laden AM in the lungs were unable to clear surfactant, leading to mortality through gas exchange interruption. While we report that *Nkrp1b*^−*/*−^ mice accrue a similar foamy phenotype in AM, AM appear to develop normally during the first two critical weeks post-partum before their decline in the following 4 weeks, and mice are otherwise completely healthy and viable. Moreover, while *Nkrp1b*^−*/*−^ AM tend to have a foamy, lipid-laden phenotype, which is most often accompanied by alveolar mucous/protein accumulation and easily detected through PAS staining^75,76^, *Nkrp1b*^−*/*−^ mice have no significant mucous/protein in the alveolar space at all. This is very likely due to the peripheral blood monocytes infiltrating the alveolar niche and differentiating into AM, thus providing the necessary metabolic capacity to prevent PAP development.

The CCR2-dependent replenishment of AM from the blood monocyte pool is consistent with previous reports that peripheral monocytes replace AM when resident AM are subjected to damage that impairs their ability to self-renew^3,5,77^. There also exist non-CCR2-mediated pathways of monocyte egress and entry into tissues, which likely contribute to the slight increase in the number of AM observed between the 12- and 21-week timepoint in *Nkrp1b*^−*/*−^*Ccr2*^−*/*−^ mice, such as through CX_3_CR1 and/or S1PR5^78,79^. While the alveolar niche supports incoming monocyte differentiation into AM^48,80^, subtle differences remain when compared to liver monocyte-derived tissue-resident AM^81^. These key differences in origin may account for the ability of *Nkrp1b*^−*/*−^ mice to survive the loss of tissue-resident AM. The incoming monocyte-derived AM, which are then able to carry out the necessary surfactant metabolism to prevent PAP-related mortality, do not appear to undergo full differentiation, exhibiting instead a subtle variation in CD11b, SIGLEC-F, and MHC II levels. Also, the presence of some lipid-laden cells in the lung at 12 weeks of age in *Nkrp1b*^−*/*−^ mice makes the function of NKR-P1B in monocyte-derived AM unclear. Given the subtlety and long manifestation time of the NKR-P1B-deficient AM phenotype, it is possible that upon reconstitution, AM undergo a constant cycle of cell death due to lipotoxicity and continuous replacement that would account for the longevity of the *Nkrp1b*^−*/*−^ mouse, as well as the small aberrations found in AM extracted from 12-week-old *Nkrp1b*^−*/*−^ mice.

The ability at steady state to self-renew with very little input from the peripheral blood monocyte population is a hallmark of AM. The disruption in NKR-P1B-mediated metabolic programming is a likely contributor, but not a sole cause of, self-renewal defects of AM. The observation that AM extracted from *Nkrp1b*^−*/*−^ mice were able to survive and proliferate ex vivo shows that the impairment in self-renewal in not permanent in *Nkrp1b*^−*/*−^ AM. However, the significant dysregulation of cell cycle genes in 2-week-old *Nkrp1b*^−*/*−^ mouse AM, as well as the decrease in Ki67 and characteristic hallmarks of S-phase arrest in BrdU staining in *Nkrp1b*^−*/*−^ AM, implies a potentially fundamental involvement of NKR-P1B in the renewal of AM^82,83^. This is further supported by the appearance of double nuclei in *Nkrp1b*^−*/*−^ AM cultured in vitro, which may reflect the consequence of persistent inflammation and DNA damage that is known to result in the formation of polyploid macrophages without cell-to-cell fusion^84,85^. Lack of NKR-P1B signaling resulting in the accumulation of toxic lipid species may be the trigger that induces chronic stress on the AM, some of which resort to a polyploid phenotype to preserve genomic stability or it could potentially be caused by a defect in machinery responsible for cytokinesis due to metabolic stress, thus leading to failure of cell division.

*Nkrp1b*^−*/*−^ AM exhibit a metabolic profile characterized by increased lipid uptake, storage, and processing pathways. The concomitant increase in the expression of *Apoe, CD36*, and various lipid processing enzymes, with the downregulation of cholesterol efflux pumps (*Abca1, Abcg1*), could explain the accumulation of lipid droplets in *Nkrp1b*^−*/*−^ AM, as reported for metabolically impaired AM^20,86^. Although GM-CSF receptor levels, phospho-STAT5 levels, and secreted alveolar GM-CSF levels remain unchanged in *Nkrp1b*^−*/*−^ mice, NKR-P1B activity may regulate the metabolic responses through *Rara* and *Rarg* and parts of the Ras pathway, which are downregulated in *Nkrp1b*^−*/*−^ AM and are necessary for metabolic and GM-CSF signaling, respectively^87–91^. If the dysregulation in this lipid uptake and processing pathways underlies the *Nkrp1b*^−*/*−^ phenotype, then the dramatic increase in PC, PG, LPC, and LPG in lipid-laden AM at 6 weeks of age is not entirely surprising, since these are the main constituents of pulmonary surfactant^11,92^, and therefore reflect a potentially increased uptake, reduced metabolism (or both) causing a backlog of unprocessed lipid. It is also, possible that accelerated fatty-acid biosynthesis contributes to the accumulation of lipids in *Nkrp1b*^−*/*−^ AM, as the level ACC1 of significantly increased in these cells compared to WT. TG and CE are known to be safe lipid storage molecules^52,93^, while excess accumulation of free cholesterol, free fatty acids, and DAG induces lipotoxicity^53,94,95^, exacerbated in *Nkrp1b*^−*/*−^ AM. While the mechanism by which NKR-P1B regulates lipid uptake requires further study, it may function through CD36^96^, which contains a signaling domain whose gene expression is enhanced by the binding of its ligand^97^ and is important for DPPC uptake by AM^49^. Likewise, our qPCR screen of NKR-P1B cross-linking on AM showed that transcription levels of *Serpinb6, Fabp5*, and *Apoe*, which were downregulated in *Nkrp1b*^−*/*−^ AM, were upregulated upon NKR-P1B engagement, indicating a direct involvement of NKR-P1B signaling in the transcriptional control of these genes. These findings paint a picture in which NKR-P1B loss contributes to both increased surfactant uptake and the dysregulation in lipid metabolism that leads to the storage of toxic lipid species that slowly impacts the viability of AM.

In sum, this report demonstrates the presence of lung-specific function for the NKR-P1B receptor in the control of key components of the homeostatic maintenance pathways and pulmonary surfactant clearance functions in AM, thus providing another piece of the puzzle of tissue-specific determinants governing resident macrophage identity factors.

## Methods

### Ethics statement

All animal experiments were conducted in accordance with protocols 19-135 and 20-084 approved by the Dalhousie University Animal Care Committee and the Canadian Council on the Use of Laboratory Animals.

### Study design

The study aimed to investigate the contribution of NKR-P1B to the lipid metabolism of AM since many of the tissue-specific determinants of macrophage metabolic programming are still unknown. We took advantage of NKR-P1B and Clr-b-deficient mice as well as soluble NKR-P1B tetramers to explore this relationship. The lack of phenocopying of Clr-b-deficient mice led us to explore potential alternatives for a tissue-specific NKR-P1B ligand which led us to utilize a mouse type-II pneumocyte cell line and Clr-g expression system to show the possibility of this ligand–receptor interaction in this model system.

### Experimental model and mouse details

C57BL/6 (Jackson Laboratories), *Nkrp1b*^−*/*−^ (previously described)^29^¸ and *Clr-b*^−*/*−^ (a generous gift from Dr. Matthew T. Gillespie)^98^ mice were bred in-house. CCR2-deficient mice were purchased from Jackson Laboratories and bred in-house. Generation of *Ccr2*^−*/*−^*Nkrp1b*^−*/*−^ double mutant mice entailed crossing CCR2-deficient mice and NKR-P1B-deficient mice. Mice produced from the second generation of this pair were screened by PCR to establish the *Ccr2*^−*/*−^*Nkrp1b*^−*/*−^ mouse which was subsequently bred in-house. Genotyping primers were Common forward (5’-GAACATCTTCCTCATCGGTC-3^’^), reverse deletion (5^’^-GCTGCTTAAGGATTCCATG-3^’^), reverse intron (5^’^-CACCTGTGTGACCTTTTGCA-3^’^). Animals were housed under specific pathogen-free conditions. All mice were maintained under a 12 h light/12 h dark cycle with free access to food and water in a temperature (22 ± 1 °C) and humidity (50 ± 5%) controlled room. Mice were fed autoclaved rodent feed and water ad libitum. Mice were age and gender-matched for experiments.

### Tissue processing and flow cytometry

Mice were euthanized, their lungs excised, and homogenized with a razor blade. The homogenate was incubated at 37 °C with 5% CO_2_ in the presence of 5 mg/mL collagenase D (Worthington Biochemical Corporation Cat# LS004188) for 1 h. The resulting homogenate was then passed through a 70 μm cell strainer (Falcon Cat#352350). The cell suspension was then subjected to a 20% percoll (GE Healthcare Cat# 17-0891-01) gradient and spun at 2000 × *g* for 30 min. The supernatant was pipetted off and ACK lysis was performed to lyse the red blood cells. The final cell suspension was resuspended in PBS, cell counts were determined, and then the surface was stained for flow cytometry. Single-cell lung suspensions were stained with anti-CD45-APC (BioLegend Cat#103112; 1:200), anti-MHC II-BV510 (BioLegend Cat#107635; 1:200), anti-SIGLEC-F-PerCP-eFluor 710 (Invitrogen Cat# 46-1702-82; 1:200), and in some experiments with anti-CD11c-FITC (eBioscience Cat#11-0114-85; 1:200), anti-NKR-P1B at 1:100 (a generous gift from Dr. Koho Iizuka, University of Minnesota), anti-Clr-b antibodies at 1:100 as described previously^28^. After gating out doublets, AM were identified as CD45^+^MHC II^mid^SIGLEC-F^+^CD11c^+^ cells with high forward and side scatter characteristics (see Supplementary Fig. 11a for gating strategy). In lavage experiments, mice were anesthetized by CO_2_ asphyxiation, the trachea exposed and then cannulated using a 20-gauge syringe wrapped in surgical tubing. Up to 1 mL of cold PBS was then flushed into the lung, withdrawn and the lavage fluid placed into a sterile 15 mL tube. This was repeated three more times. The lavaged AM were then used for downstream experiments. Cell isolation from the spleen was conducted by excising the spleen from the mouse and crushing it between two microscope slides. The resulting cell suspension was subjected to ACK lysis after which it was resuspended in 10 mL PBS and used for flow cytometry. For isolation of Kupffer cells, mouse livers were mashed through a 70 μm cell strainer, spun down, resuspended in 37% percoll, and centrifuged at 700×*g* for 30 min. Afterward, the cells were subjected to ACK lysis, resuspended in 100 μL, and stained for flow cytometry. Large and small peritoneal macrophages were extracted by injecting cold PBS into the peritoneal cavity, gently massaging the area to dislodge any cells, and then removing the PBS into a tube. This process was repeated four times. Cells were then spun down, resuspended in 100 μL, and stained for flow cytometry. Experiments with anti-phospho-p38 (BioLegend Cat# 690203; 1:50) staining were performed on lavaged AM from 2-week-old WT and *Nkrp1b*^−*/*−^ mice. NOS2 levels were determined by staining lavaged AM from 2-week-old WT and *Nkrp1b*^−*/*−^ mice with anti-NOS2 antibody (BioLegend Cat# 696805; 1:50). For glucose uptake analysis, 2-(*N*-(7-Nitrobenz-2-oxa-1,3-diazol-4-yl)Amino)-2-Deoxyglucose (2-NBDG) from (Cayman Chemical Cat#11046) was prepared as a 30 mM stock solution by dissolving in DMSO. 2-NBDG or vehicle was added to a final concentration of 25 μM to AM obtained from 2-week-old WT and *Nkrp1b*^−*/*−^ mice and left to incubate with the AM for 20, 40, and 60 min. Cells were then washed once with FACS buffer, stained for viability, and immediately acquired on a flow cytometer without fixation. MFIs were used to analyze 2-NDBG uptake. For Tetramethyl rhodamine ethyl ester (TMRE) staining 1 mM TMRE (Thermo Fisher Cat#T669) stock solutions were prepared by dissolving in DMSO. AM obtained from 2-week-old WT and *Nkrp1b*^−*/*−^ mice were stained with 50 nM TMRE for 20 min and then immediately acquired on a flow cytometer. For mitochondrial mass analysis, lavaged AM obtained from 2-week-old WT and *Nkrp1b*^−*/*−^ mice were stained using MitoTracker Red CMXRos from Thermo Fisher (Cat#M7512) as per manufacturer’s instructions. Cellular ROS was analyzed on lavaged AM obtained from 2-week-old WT and *Nkrp1b*^−*/*−^ mice using DCFDA/H2DCFDA cellular ROS assay kit from Abcam (Cat#ab113851) as per manufacturer’s instructions. FlowJo 10.0.6 software was used to analyze FACS data.

### Histology and immunofluorescence

For Periodic-acid Schiff (PAS) staining, formalin-fixed paraffin-embedded tissues were cut at 5 μm using a microtome, deparaffinated, and stained with a PAS kit as per manufacturer’s instructions (Abcam Cat#ab150680). For ORO staining, a stock solution was purchased from Sigma-Aldrich (Cat#01391). For some experiments, lungs were inflated with OCT and 20% sucrose and snap-frozen in liquid nitrogen. Frozen sections were cut at 10 μm using a cryostat then air-dried for 30 min. ORO staining was performed by fixing the sections with three washes of 60% isopropanol, the incubating them with a working concentration (66%) of ORO solution for 15 min. Slides were then washed quickly with 60% isopropanol, quickly stained with Mayer’s hematoxylin to highlight nuclei, and then mounted using aqueous mounting media (Vectorlabs Cat#H-5501). Immunofluorescence staining was conducted on 10 μm frozen lung sections. After cutting sections were fixed in acetone for 13 min and left to dry overnight. Sections were blocked with normal horse serum for 1 h and then incubated with anti-SIGLEC-F antibody (Invitrogen Cat#14-1702-82) overnight. Nuclei were highlighted using DraQ5 (Abcam Cat#ab108410) by incubating the sections for 10 min with a 1:1000 dilution of DraQ5 in PBS. The sections were then mounted and visualized on a Zeiss 550 confocal microscope. For immunofluorescence staining of phagolysosomes, lavaged AM from 6-week-old WT and *Nkrp1b*^−*/*−^ mice were stained with anti-Rab7 (Abcam Cat#ab137029; 1:50) and anti-CD107a/Lamp-1 (Biotechne Cat#MAB4320-SP; 1:50). Cells were mounted in DAPI containing fluorescent mounting medium and imaged on a Leica SP8 confocal microscope. Colocalization of Rab7 and Lamp-1 staining was performed using Coloc2 plugin for ImageJ software^99^.

### Electron microscopy

Mice aged 2, 6, and 12 weeks were lavaged as described above. AM from four mice was pooled together to obtain enough cells for one biological replicate. The cell pellet was fixed in 2.5% glutaraldehyde solution and then processed using the 1% osmium tetroxide/uranyl acetate method. Samples were cut using a Reichert-Jung ultracut E ultramicrotome, viewed on a JEOL JEM 1230 transmission electron microscope, and images captured using a Hamamatsu ORCA-HR digital camera.

### Lipid uptake assays and NKR-P1B cross-linking

Lipid uptake assays were performed on AM from 2-week-old WT and *Nkrp1b*^−*/*−^ mice by lavage as described above. AM from three mice was pooled together for each replicate per independent experiment. Nitrobenzofurazan-labeled dipalmitoyl-phosphatidylcholine (NBD-PC) and phosphatidylglycerol (NBD-PG) (Avanti BioLipids Cat# 810131, 801064 respectively). NBD-PC and NBD-PG were resuspended in 90% ethanol to a concentration of 4 mg/mL. For the assay, a final lipid concentration of 20 μg was applied to lavaged AM resuspended in 100 μL of DMEM with no supplements and then allowed to incubate for 10, 20, and 40 min. Cells were then washed once with FACS buffer, stained for viability, and immediately acquired on a flow cytometer without fixation. MFIs were used to analyze lipid uptake. In some experiments, lavaged AM were first blocked using normal mouse serum, stained with biotinylated anti-NKR-P1 B (0.05 mg/mL) antibody, and then cross-linked in suspension with streptavidin (0.02 mg/mL) for 1, 3, 6, or 16 h and then analyzed for lipid uptake as described above. Experiments that evaluated NBD-PC uptake in the presence of 35 U/mL GM-CSF from Cell-Signal (Cat#7043S) were performed on lavaged AM from 2-week-old WT and *Nkrp1b*^−*/*−^ mice.

### Bead phagocytosis assay

Phagocytosis of fluorescently labeled beads was analyzed on lavaged AM obtained from 2-week-old WT and *Nkrp1b*^−*/*−^ mice using the Cayman Chemical phagocytosis assay kit (Cat#500290) as per the manufacturer’s instructions. Surface fluorescence was quenched using trypan blue just prior to data acquisition by flow cytometry.

### Glucose uptake assay

For glucose uptake analysis, 2-(*N*-(7-Nitrobenz-2-oxa-1,3-diazol-4-yl)Amino)-2-Deoxyglucose (2-NBDG) from (Cayman Chemical Cat#11046) was prepared as a 30 mM stock solution by dissolving in DMSO. 2- NBDG or vehicle was added to a final concentration of 25 μM to AM obtained from 2-week-old WT and *Nkrp1b*^−*/*−^ mice and left to incubate with the AM for 20, 40, and 60 min. Cells were then washed once with FACS buffer, stained for viability, and immediately acquired on a flow cytometer without fixation. MFIs were used to analyze 2-NDBG uptake.

### Extracellular oxygen consumption assay

AM lavaged from 2-week-old WT and *Nkrp1b*^−*/*−^ were analyzed for O_2_ consumption kinetics by the extracellular oxygen consumption assay kit (Abcam Cat#ab197243) as per the manufacturer’s instructions. Oxygen consumption data were acquired on a CLARIOstar high-performance plate reader (BMG Labtech). For glucose uptake analysis, 2-(*N*-(7-Nitrobenz-2-oxa-1,3-diazol-4-yl)Amino)-2-Deoxyglucose (2-NBDG) from (Cayman Chemical Cat#11046) was prepared as a 30 mM stock solution by dissolving in DMSO. 2- NBDG or vehicle was added to a final concentration of 25 μM to AM obtained from 2-week-old WT and *Nkrp1b*^−*/*−^ mice and left to incubate with the AM for 20, 40, and 60 min. Cells were then washed once with FACS buffer, stained for viability, and immediately acquired on a flow cytometer without fixation. MFIs were used to analyze 2-NDBG uptake. For Tetramethyl rhodamine ethyl ester (TMRE) staining 1 mM TMRE (Thermo Fisher, Cat# T669) stock solutions were prepared by dissolving in DMSO. AM obtained from 2-week-old WT and *Nkrp1b*^−*/*−^ mice were stained with 50 nM TMRE for 20 min and then immediately acquired on a flow cytometer. For mitochondrial mass analysis, lavaged AM obtained from 2-week-old WT and *Nkrp1b*^−*/*−^ mice were stained using MitoTracker Red CMXRos from ThermoFisher (Cat#M7512) as per manufacturer’s instructions. Cellular ROS was analyzed on lavaged AM obtained from 2-week-old WT and *Nkrp1b*^−*/*−^ mice using DCFDA/H2DCFDA cellular ROS assay kit from Abcam (Cat#ab113851) as per the manufacturer’s instructions.

### SCENITH

SCENITH assay was performed using the protocol, compounds, and concentrations described in Arguello et al. Cell Metabolism, 2020^54^. AM were lavaged and resuspended in RPMI-1640 and then treated with Harringtonine, Oligomycin-A, 2-Deoxglucose, 2-Deoxyglucose + Oligomycin-A simultaneously or vehicle control for 15 min. After 15 min, Puromycin was added after 15 min and incubated with AM for a further 30 min (total incubation time is therefore 45 min). Afterward, the AM were washed, stained for viability, and then stained for puromycin using the FOXP3 Fix/Perm staining kit (BioLegend, #421403) and the anti-puromycin 647 (12D10) antibody (MilliporeSigma, MABE343-AF647; 1:50) or an IgG2a, κ Isotype Control Alexa Fluor 647 antibody (BDBiosciences, #558053; 1:100). All samples were acquired by flow cytometry on a BD-LSR Fortessa cytometer.

### Met-Flow analysis

AM isolated from 2-week-old mouse lavage fluid was used for the Met-flow analysis. AM were fixed and permeabilized using eBioscience FoxP3/Transcription factor staining buffer set (Invitrogen, Cat#00-5523-00) as per the manufacturer’s instruction. AM were stained for 1 transmembrane and 8 different intracellular metabolic targets, which are Glucose transporter member 1 (GLUT1), Glucose-6-phosphate dehydrogenase (G6PD), Hexokinase 1 (HK1), Carnitine palmitoyl-transferase 1A (CPT1A), Acetyl-CoA carboxylase alpha (ACC1), Isocitrate dehydrogenase 2 (IDH2), ATP synthase F1 subunit alpha (ATP5A), Argininosuccinate synthase 1 (ASS1), and Peroxiredoxin 2 (PRP), respectively, as described in P.J. Ahl et al.^55^. The antibodies used for this assay are Rabbit mAb anti-GLUT1-Alexa Flour 405 (Abcam, Cat#ab210438; 1:25), CoraLite 488-conjugated anti-G6PD mouse mAb (Proteintech, Cat#CL488-66373; 1:25), rabbit mAb anti-HK1-Alexa Flour 647 (Abcam, Cat#ab197864; 1:25), mouse mAb anti-CPT1A- Alexa Flour 488 (Abcam, Cat#ab171449; 1:25), rabbit mAb anti-ACC1-Alexa Flour 488 (Abcam, Cat#ab203994; 1:25), rabbit mAb anti-IDH2 PE (Abcam, Cat#ab212122; 1:25), rabbit mAb anti-ATP5A-Alexa Flour 594 (Abcam, Cat#ab216385; 1:25), rabbit mAb anti-ASS1-PE (Abcam, Cat#ab210451; 1:25), and rabbit mAb anti-PRP-PE(Abcam, Cat#ab197536; 1:25). The samples were then acquired on a BD-LSR Fortessa flow cytometer.

### In situ hybridization

In situ hybridization was performed using the Roche DIG RNA labeling kit SP6/T7 (Roche Cat#11175025910) as described previously by our research group^33^, with some minor modifications. The hybridization temperature of the Clr-g probe during the overnight hybridization step was 56 °C. The slides were then scanned on a Leica Aperio slide scanner and visualized using the Leica Aperio-ImageScope software.

### In situ tetramer staining

NKR-P1B Tetramer assembly was conducted by the addition of PE-labeled streptavidin at a molecular ratio of 4:1 biotin monomer:streptavidin. 1/10th of the PE-labeled streptavidin was added at a 15-min interval at 4 °C. NKR-P1B monomers were generated as described previously^100^. Frozen lung sections were cut at 10 μm and left to air-dry for at least 3 h. Native biotin was blocked using the BioLegend avidin-biotin blocking system (Cat#927301) as per the manufacturer’s instructions. Sections were subsequently blocked using rodent M block (ThermoFisher Cat#5083262) for 2 h. Primary antibodies against CD45 (1:100) (BioLegend Cat#103101; 1:200) and PSPTC (1:500) (Abcam Cat#ab90716) and 1.1 μg of NKR-P1B tetramers were applied to the sections and incubated overnight at 4 °C. The next day, sections were washed once in PBS and fixed using 4% paraformaldehyde (PFA) for 12 min and washed three times in PBS. The sections were then incubated for 2 h with 1:100 anti-PE antibody (BioLegend Cat#408101;1:200). The sections were washed and incubated with anti-rat IgG-AlexaFluor 488 (Invitrogen Cat#A-11001; 1:200), anti-mouse IgG-AlexaFluor 555 (BioLegend Cat#405324; 1:200), and anti-goat IgG-Cy5 (Invitrogen Cat#A-10523; 1:200) secondary antibodies for 1 h at room temperature. Sections were then washed three times in PBS and mounted using Vectashield (Vector Labs Cat#H-1200-10), DAPI containing fluorescent mounting medium. Sections were imaged on a Leica SP8 confocal microscope.

### *S. pneumoniae* infections

Two, six, and 12-week-old mice age and sex-matched mice were infected intranasally with 730 CFU/g bodyweights of *S. pneumoniae* encapsulated serotype III (ATCC 6303). Mice were monitored for up to 7 days and sacrificed when reached appropriate endpoints which included either 20% weight loss or a combination of external observable factors (labored breathing, low response to stimulus, ruffled fur, hunched posture, etc.). For colony-forming unit (CFU) counts, mice were sacrificed at day 3 post-infection and lavaged as described previously. The lavage fluid was then plated onto fresh sheep blood agar plates and the alpha-hemolytic colonies, indicating *S. pneumoniae*, were counted and CFUs determined.

### In vivo BrdU assay

Two-week-old male and female mice were injected with 1 mg of BrdU intraperitoneally. 24 h later mice were sacrificed and lavaged as described previously to obtain the AM. The cells were then processed using the BioLegend Phase-Flow kit (Cat#370704) as per the manufacturer’s instruction with no modification. The samples were then run on a BD-Fortessa flow cytometer and analyzed for cell cycle status.

### Pravastatin treatment

Three-week-old *Nkrp1b*^*+/+*^*Ccr2*^−*/*−^ and *Nkrp1b*^*−/*^*Ccr2*^−*/*−−^ male or female mice were given either regular water or water supplemented with pravastatin calculated to deliver 10 μg/g bodyweight of the drug each day. The mice were treated for two weeks after which the lungs were extracted and frozen at −80 °C and processed for ORO staining as described above.

### NK cell depletion

NK cell depletion was achieved by intraperitoneally injecting 6-week-old male or female WT mice with either 200 μg of anti-NK1.1 antibody, derived from the PK136 hybridoma or mock saline injection (*n* = 8 per treatment group). Depletion was maintained through injection of 100 μg of anti-NK1.1 mAb every 48 h. Throughout the anti-NK1.1 depletion treatment, mice were infected with *S. pneumoniae* as described above.

### Cell lines and transfections

The full coding sequence for NKR-P1B was isolated from mouse cDNA and inserted into the pLJM1 vector (Addgene 91980). Lentiviral assembly was conducted in HEK293T cells (ATCC CRL3216) by PEI transfection with the pLJM1 vector containing the NKR-P1B sequence or an empty pLJM1 vector, psPAX2 viral packaging vector, and the pMD2G viral envelope vector. Cell supernatants were harvested 48 h post-transfection and lentiviral stocks were kept at −80 °C until further use. MLE-12 cells obtained from ATCC (CRL-2110) were grown on coverslips in HITES medium. MLE-12 cells were then transfected with either an empty PcDH vector (Addgene 72299) or PcDH vector containing the sequence for C57BL/6 mouse Clr-g gene using FuGene (Promega Cat# E2311). Transfection efficiency was verified by GFP expression as observed on an immunofluorescent microscope. After 48 h the media and transfection reagent was removed, and cells were fixed with 4% PFA for 12 min then stained with NKR-P1B tetramers as described earlier.

### RNA sequencing

AM were extracted from 2-week-old mouse lungs as described above and sorted on a BD FACS ARIA II cell sorter achieving an average of 98% purity. RNA from the sorted AM was extracted using Ribozol (VWR Cat#97064-952) in conjunction with ZymoResearch RNA clean-up and concentrator kit (Zymogen Cat#R1016) to achieve the required purity and quantity of RNA for sequencing. AM from 6 mice was pooled together to represent one biological replicate. The RNA sequencing was performed by Genome Quebec (McGill University). FASTQ files were uploaded to the Galaxy web platform and analyzed using the public usegalaxy.org server. Briefly, Trimmomatic v0.39 was used to cut out primer sequences, followed by cufflinks assisted sequence alignment to reference genome and significant up or downregulated genes calculated using the Cuffdiff v2.2.1.

### RT-PCR

Total lungs from 6-week-old WT and *Clr-b*^−*/*−^ mice were processed as described above and a CD45^−^ CD31^−^ CD104^−^ EPCAM^hi^ type-II pneumocytes were FACS sorted using, anti-CD45-FITC (BioLegend Cat#103101), anti-CD326 (EpCAM)-PE (BioLegend Cat#118205; 1:200), anti-CD31-FITC (BioLegend Cat#102405; 1:200), and anti-CD014-FITC (BioLegend Cat#123605; 1:200) antibodies into Ribozol (VWR). The RT-PCR primers for *Clr-b* RT-PCR were *Clr-b* forward (5’-ACTCAGCTCCTCAGCTCTGA-3’) and *Clr-b* reverse(5’-GGCTAAAAAGCGTCTCTTGG-3’), and the primers for *Clr-g* RT-PCR were: *Clr-g* forward (5’-AGATTGCTTGGAGACAGGAG-3’) and *Clr-g* reverse (5’-GAAGAGTCTCTTGGTAAGTG-3’)^33^

### RT-qPCR

AM were extracted from 2-week-old WT and *Nkrp1b*^−*/*−^ mice, or WT AM following NKR-P1B crosslinking as described, and total RNA was isolated using Ribozol (VWR Cat#97064-952) in conjunction with an RNA clean-up and concentrator kit (Zymo Research; cat # R1016). cDNA was synthesized using RevertAid First Strand cDNA Synthesis kit (Thermo Scientific; cat # K1622). SYBR-green-based (qPCR) was performed on 0.1–0.5 μg of cDNA using NEB Luna Universal qPCR Master Mix (NEB; cat # M3003S/L/X/E). Data was acquired on a BioRad CFX96 Real-Time PCR analyzer. Primers used to amplify target genes *CD36*, *CD63*, *Ldlr*, *Nceh*, *ABCA1*, *ABCG1*, *Serpinb6*, *Rara*, *Fabp5*, *ApoE* and *Psap* are listed in Table 1 (see Supplementary Data 1).

### ELISA

ELISA for GM-CSF was obtained from BioLegend (Cat#432207). Two-week-old mice were lavaged as described above with minor modifications. The lungs were lavaged once using 500 μL of PBS. Samples were processed as per the manufacturer’s instructions.

### Phospho-STAT5 Assay

AM isolated from 2-week-old mouse lavage fluid as described earlier were fixed with 2% PFA for 25 min at 4 °C and permeabilized using 100% ice-cold methanol for 45 min on ice. Finally, AM were stained for Tyr694-phosphorylated STAT5 (pSTAT5) with an anti-pSTAT5-PE antibody (Invitrogen, Cat#12-9010-42; 1:50) or IgG1, k-PE Isotype Control antibody (eBioscience12-4714-71; 1:100) for 1 h at room temperature. As a positive control, thymocytes from 6-week-old C57BL/6J mouse thymus extractions were collected, resuspended in ACK lysis buffer to lyse the red blood cells, and then incubated with recombinant IL-2 for 15 min at 37 °C before being processed for flow staining for pSTAT5 (Supplementary Fig. 2q). The samples were acquired on a BD-LSR Fortessa cytometer.

### Chemokine dot plot

500 μl of BAL and 200 μl of serum from 6-week-old WT and *Nkrp1b*^*−/−*^ mice were applied to a Proteome Profiler Mouse Chemokine Array Kit from R&D Systems (Cat#ARY020) and processed according to manufacturer’s directions.

### Lipidomic analysis

AM were collected by lavage from 6-week-old mice as described above. AM from 5 mice was pooled to represent one biological replicate. The lavage fluid was spun, the supernatant removed, and the cell pellet frozen at −80 °C for analysis. Analysis was performed at the Laboratory of Genetic Metabolic Diseases, the University of Amsterdam as previously described^101^.

### Statistical analysis

Statistical significance was determined by Student’s *t*-test where applicable, with a cut-off *p-*value of 0.05. Other experiments, where appropriate utilized a two-way ANOVA or one-way ANOVA analysis with Tukey’s or Šídák post hoc correction for multiple comparisons. Significance in survival experiments was determined using the log-rank test with a cut-off *p*-value of 0.05. Significance differences in oxygen consumption over time were determined by linear regression analysis. Graphs and statistics were generated using GraphPad Prism v9.

### Reporting summary

Further information on research design is available in the Nature Portfolio Reporting Summary linked to this article.

## Supplementary information

Supplementary Information

Description of Additional Supplementary Files

Supplementary Data 1

Reporting Summary

## Data Availability

The RNA-seq data generated in this study have been deposited in the NCBI Gene Expression Omnibus database under primary accession code GSE214867. All other data are available in the article and its Supplementary files or from the corresponding author upon request. Source data are provided with this paper.

## Source data

Source Data
