# Supplementary material for: Alveolar macrophage metabolic programming via a C-type lectin receptor protects against lipo-toxicity and cell death
Source: Nat Commun. 2022 Nov 25;13:7272. doi: 10.1038/s41467-022-34935-w (PMC9700784; doi:10.1038/s41467-022-34935-w)
Supplement: Supplementary file 1 — Supplementary Information [file 41467_2022_34935_MOESM1_ESM.pdf]

Supplementary Information

Metabolic Programming Via a C-type Lectin Receptor Protects Alveolar Macrophages From Foam Cell Formation and Death by Lipotoxicity

Michal Scur, Ahmad Bakur Mahmoud, Sayanti Dey, Farah Abdalbarri, Iona Stylianides, Daniel Medina-Luna, Gayani S. Gamage, Aaron Woblistin, Alexa N. M. Wilson, Haggag S. Zein, Ashley Stueck, Andrew Wight, Oscar A. Aguilar, Francesca Di Cara, Brendon D. Parsons, Mir Munir A. Rahim, James R. Carlyle, and Andrew P. Makrigiannis*

*Corresponding author: andrew.makrigiannis@dal.ca

This file includes:

Supplementary Figures 1-10

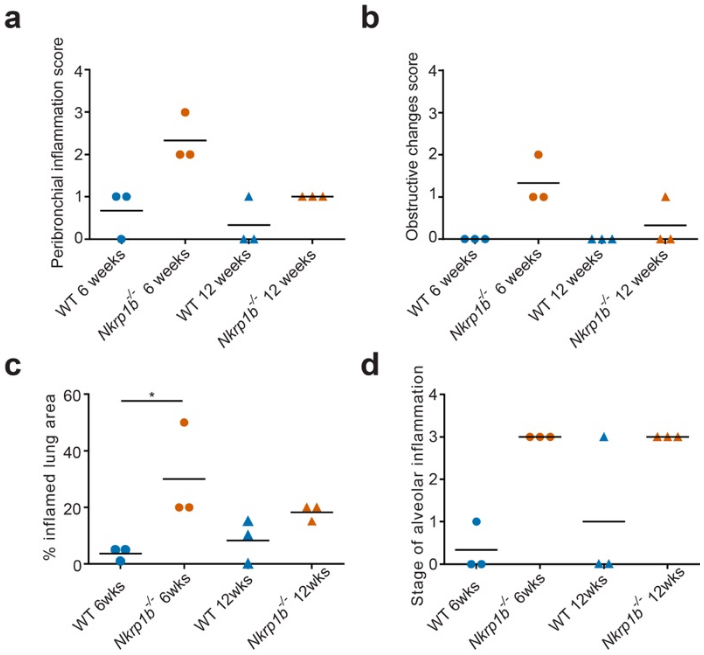

Supplementary Figure 1. Histological interpretations of pneumococcal infected *Nkrp1b*$^{-/-}$ mice. (**a** - **d**) Pathological interpretations of H/E-stained mouse lung sections extracted from 6- and 12-week-old WT and *Nkrp1b*$^{-/-}$ mice 3 days post-infection with *S. pneumoniae*. Scoring of peribronchial and alveolar inflammation, percent inflamed area, and obstructive changes. (n = 3 mice per genotype per timepoint). Statistical significance was determined by one-way ANOVA, $p = 0.0417$. In all graphs, WT is indicated in blue and *Nkrp1b*$^{-/-}$ in orange. Source data are provided as a Source Data file.

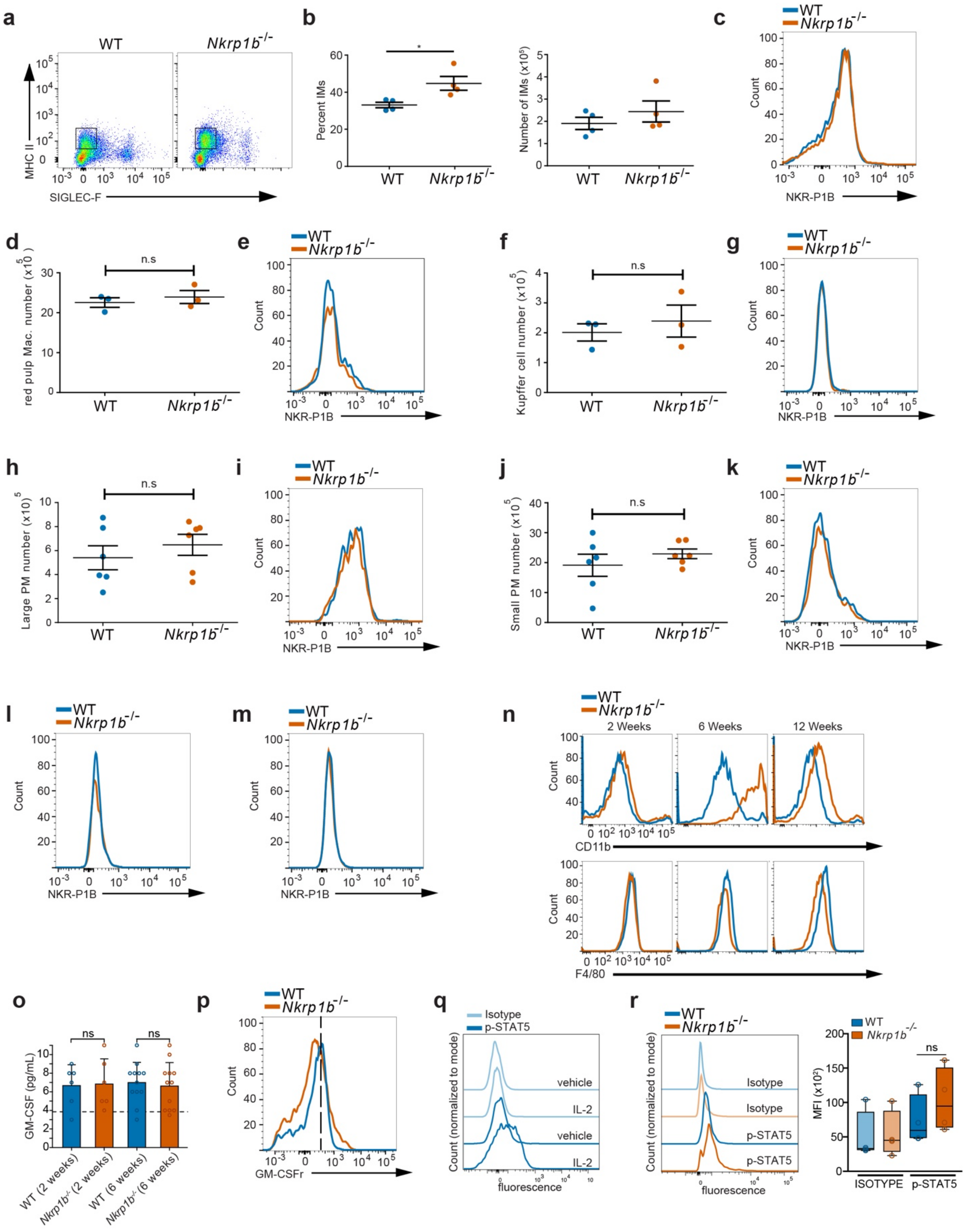
a
WT
Nkrp1b-/-
MHC II
SIGLEC-F
b
Percent IMs
Number of IMs (x10^5)
WT
Nkrp1b-/-
c
Count
NKR-P1B
d
red pulp Mac. number (x10^5)
n.s
e
f
Kupffer cell number (x10^5)
g
h
Large PM number (x10^5)
i
j
Small PM number (x10^5)
k
l
m
n
2 Weeks
6 Weeks
12 Weeks
CD11b
F4/80
o
GM-CSF (pg/mL)
ns
WT (2 weeks)
Nkrp1b-/- (2 weeks)
WT (6 weeks)
Nkrp1b-/- (6 weeks)
p
GM-CSFr
q
Isotype
p-STAT5
vehicle
IL-2
Count (normalized to mode)
fluorescence
r
MFI (x10^2)
ISOTYPE
p-STAT5

Supplementary Figure 2. Analysis of major tissue-resident macrophage populations and GM-CSF signaling. (**a**) Flow cytometry dot plots of WT and *Nkrp1b*$^{-/-}$ lung interstitial macrophages in 6-weeks-old mice. Images are representative of 2 independent experiments. (**b**) Quantifications of cell percentages and absolute numbers of interstitial macrophages (IM) shown in (a). (n = 4 mice per genotype.) *p = 0.042 (**c**) NKR-P1B surface expression on WT and *Nkrp1b*$^{-/-}$ interstitial macrophages. (**d** and **e**) NKR-P1B surface expression and cell numbers of WT and *Nkrp1b*$^{-/-}$ splenic red pulp macrophages (n = 3 mice per genotype), (**f** and **g**) liver Kupffer cells (n = 3 mice per genotype), (**h** and **i**) large peritoneal macrophages (PM) (n = 6 mice per genotype), and (**j** and **k**) small peritoneal macrophages (n = 6 mice per genotype), in 6-week-old mice as determined by flow cytometric phenotyping. (**l** and **m**) NKR-P1B expression on Ly6C+ and Ly6C- peripheral blood monocytes as determined by flow cytometry. Histogram is representative of 3 independent experiments. (**n**) Expression profile of CD11b and F4/80 on AM obtained from lungs of 2-, 6- and 12-week-old WT and *Nkrp1b*$^{-/-}$ mice. Histograms are representative of 3 independent experiments. (**o**) GM-CSF ELISA assay of WT and *Nkrp1b*$^{-/-}$ 2- and 6-week-old mice lavage fluid. (n = 3 independent experiments). Experiments performed in duplicate. (**p**) Expression of AM GM-CSFr in 2-week-old *Nkrp1b*$^{-/-}$ mice. Histogram is representative of 3 independent experiments. (**q**) Representative flow cytometry histograms of isotype or phospho-STAT5 antibody stained thymocytes from 6-week-old WT mice following treatment with IL-2 or vehicle. (**r**) Representative flow cytometry histograms of isotype control antibody or anti-phospho-STAT5 antibody staining of AM obtained from 2-week-old WT and *Nkrp1b*$^{-/-}$ mice. Quantification of mean fluorescence intensities of phospho-STAT5 staining is shown. Boxplots indicate mean, IQR, and data minimum and maximum whiskers. (n = 4 mice per genotype). Data in (b, d, f, h, j) shows mean ± SEM, and (o) shows mean ± SD. Statistical significance in (b, d, f, h, j, o, and r) was

determined by an unpaired, two-tailed Student's *t*-test. In all graphs and histograms WT is blue and *Nkrp1b*$^{-/-}$ is orange. Source data are provided as a Source Data file.

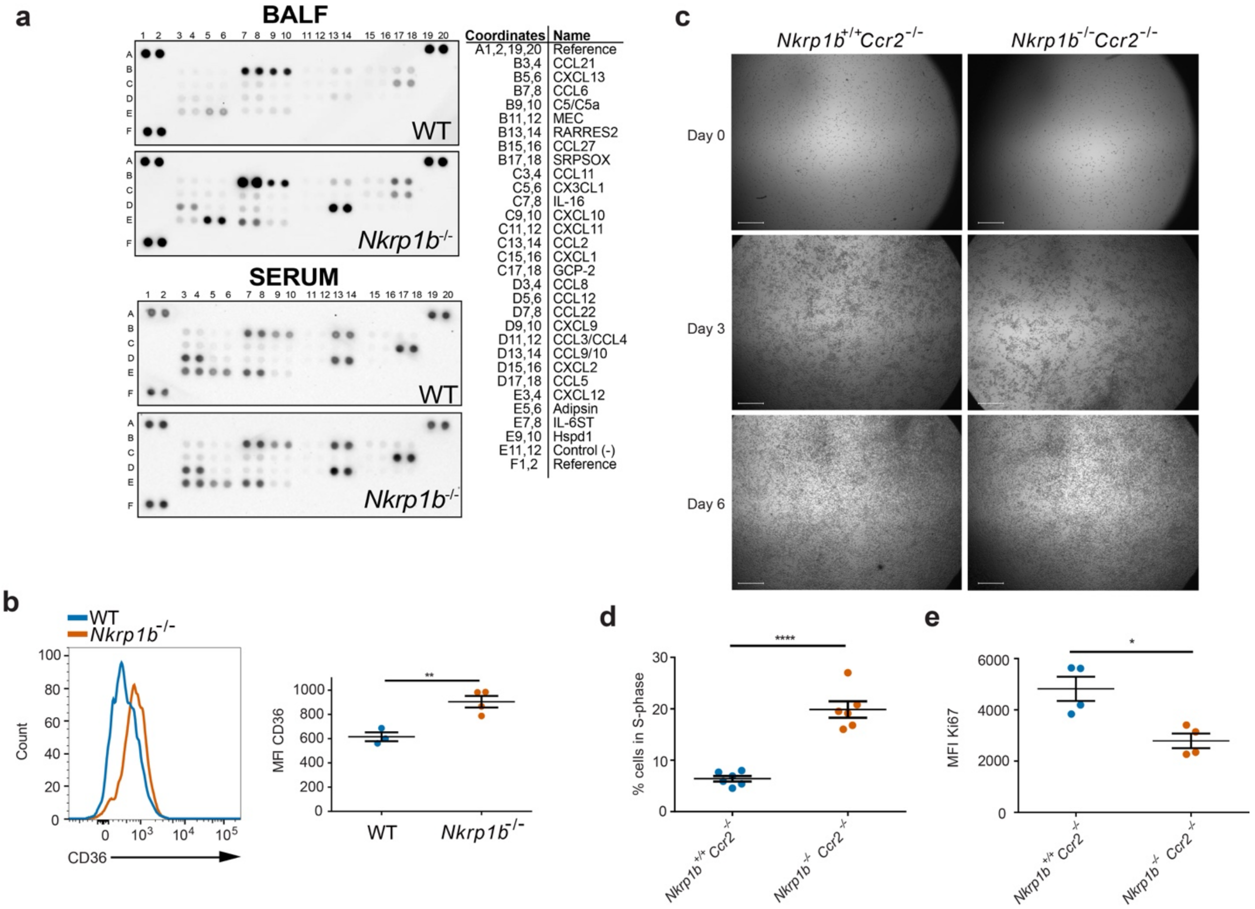

Supplementary Figure 3. Profiling of chemokines in *Nkrp1b*$^{-/-}$ mouse lungs and blood, CD36 and Ki67 levels in *Nkrp1b*$^{-/-}$ AM, and in vitro proliferation. (**a**) Dot blot chemokine array performed on WT and *Nkrp1b*$^{-/-}$ lavage fluid and serum derived from 6-week-old mice. pairs of dots in the corners of the blot represent orientation points. Positive control dots (proteins complement factor D, GP130, HSP60, which are commonly found in serum, cell lysates, etc). All chemokines, controls, and orientation dots are present on the blot in duplicate. (**b**) Expression profile of CD36 on AM obtained from 2-week-old WT and *Nkrp1b*$^{-/-}$ mice. Histogram is representative of 3 independent experiments. MFI quantifications of CD36 expressions on AM

obtained from WT (blue) and *Nkrp1b*$^{-/-}$ (orange) 2-week-old mice. Data presented as mean ± SEM. (n = 3) and (n = 4) respectively. (**c**) Image captures of lavaged AM isolated from 2-week-old *Nkrp1b*$^{+/+}$*Ccr2*$^{-/-}$ and *Nkrp1b*$^{-/-}$*Ccr2*$^{-/-}$ mice. Scale bar represents 500 µm. AM were cultured in vitro in presence of GM-CSF. Images are representative of 3 independent experiments. (**d**) Quantifications of AM in S-phase ex vivo. Data presented as mean ± SEM. (n = 6 mice per genotype). (**e**) MFI quantifications of Ki67 expressions on AM obtained from 2-week-old *Nkrp1b*$^{+/+}$*Ccr2*$^{-/-}$ (blue) and *Nkrp1b*$^{-/-}$*Ccr2*$^{-/-}$ (orange) mice. Data presented as mean ± SEM. (n = 4 mice per genotype). Significance in (b, d, and e) was determined by unpaired, two-tailed Student's t-test where *$p < 0.05$ and **$p < 0.01$. Source data are provided as a Source Data file.

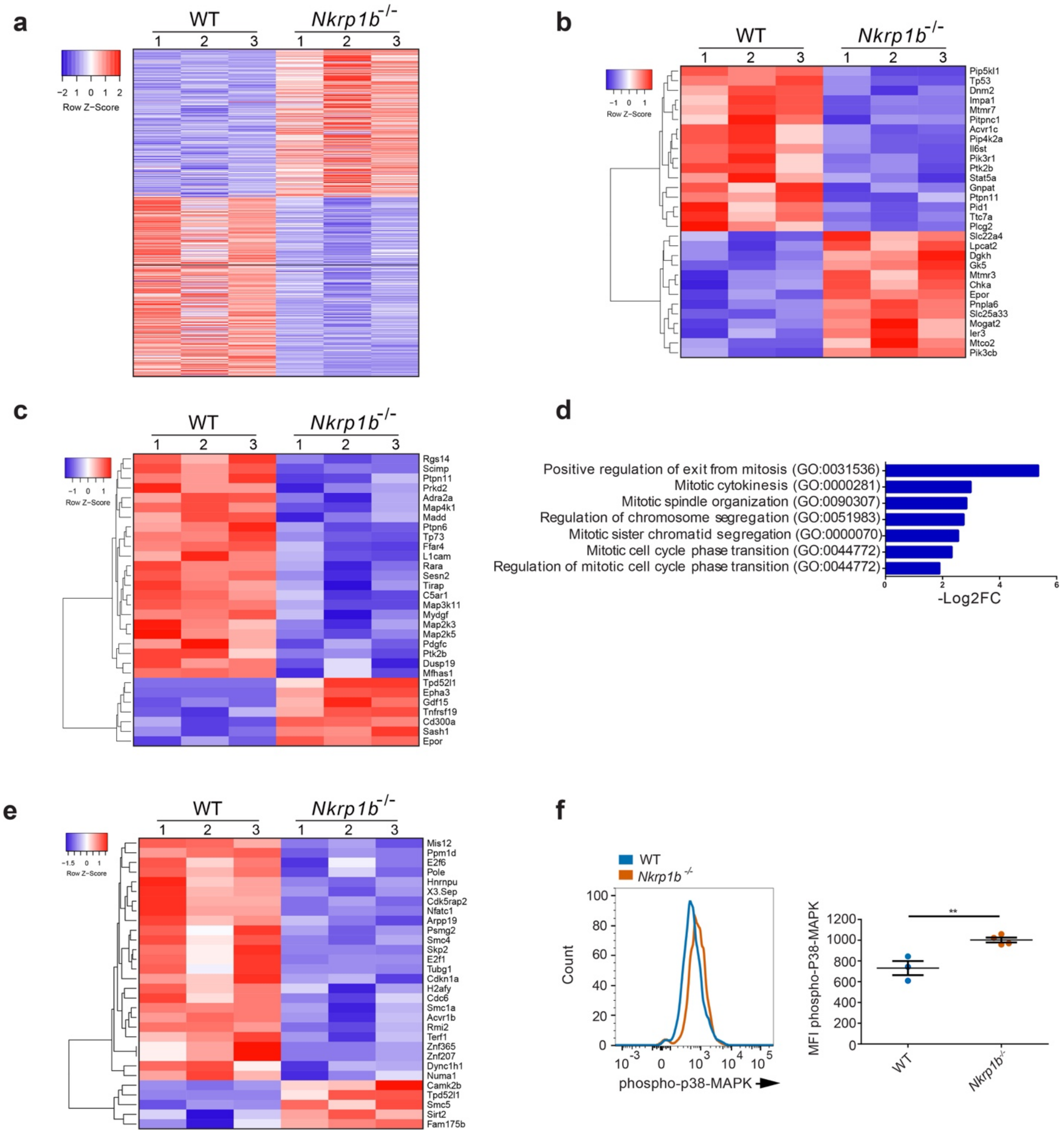

Supplementary Figure 4. Altered cell cycle processes, lipid metabolism, and MAPK/ERK pathways reveal signaling dysfunction in *Nkrp1b*$^{-/-}$ AM. (**a**) Heatmap showing all significantly dysregulated genes as determined by Cuffdiff analysis of RNA-seq data. Data presented as Z-scores of Log2 fold change. (**b**) Top 30 dysregulated genes related to lipid metabolism and processing as determined by RNA-seq analysis. Data presented as Z-scores of Log2 fold change.

(**c**) Top 30 dysregulated genes related ERK and P38MAPK kinase activity as determined by RNA-seq analysis. Data presented as Z-scores of Log2 fold change. (**d**) Gene ontology pathways related to cell cycle enriched (-Log2 fold change) in AM obtained from *Nkrp1b*$^{-/-}$ mice at 2 weeks of age as determined by RNA-seq analysis. (**e**) Top 30 dysregulated genes related cell cycle as determined by RNA-seq analysis. Data presented as Z-scores of Log2 fold change. (**f**) Histograms showing the amount of phosphorylated p38-MAPK in WT (blue) and *Nkrp1b*$^{-/-}$ (orange) AM taken from 6-week-old mice. Plot representative of 3 independent experiments. MFI of phosphorylated p38-MAPK in WT and *Nkrp1b*$^{-/-}$ AM. (n = 3) and (n = 5) for WT and *Nkrp1b*$^{-/-}$ respectively. Data presented as mean ± SEM. Statistics represent an unpaired, two-tailed Student's *t*-test where * $p < 0.05$ and ** $p < 0.01$. Source data are provided as a Source Data file.

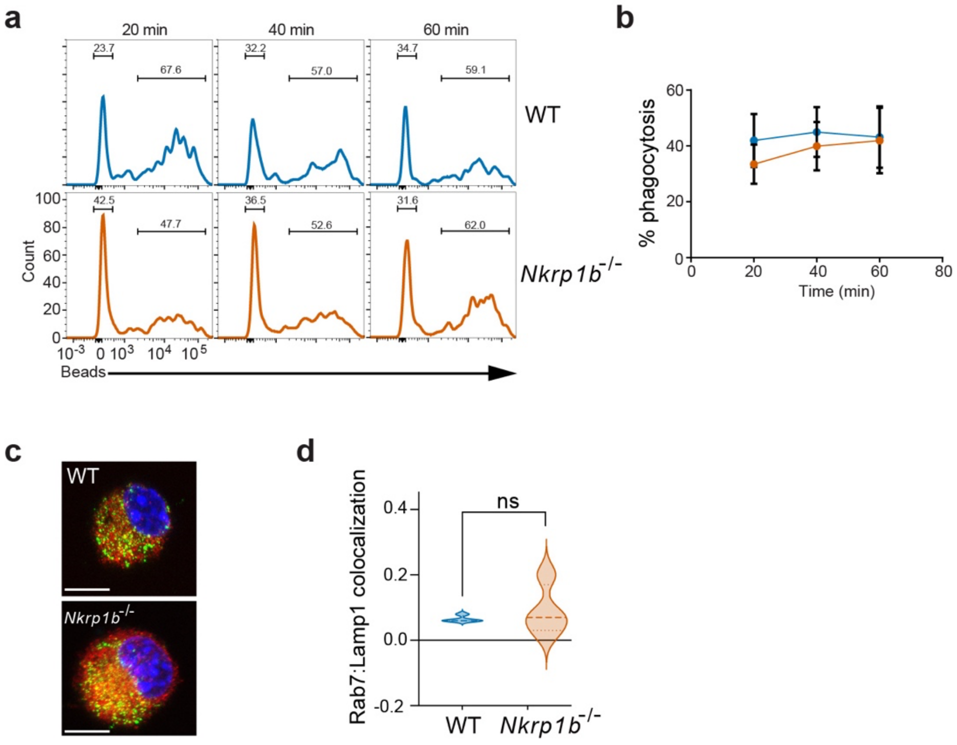

Supplementary Figure 5. Quantification of NKR-P1B role in phagocytosis and phagolysosome formation. (**a**) representative histograms of AM derived from 2-week-old WT and *Nkrp1b*$^{-/-}$ mice subjected to bead phagocytosis assay after 20, 40, or 60-minute incubation with labeled beads. Peak further down the x-axis denotes AM which have taken up the fluorescently labeled beads. (**b**) quantification of % phagocytosis as shown in (S5a), no significant differences were detected. Surface fluorescence was quenched using trypan blue before acquisition. Data is representative of 3 independent experiments. (*n* = 5). Data presented as mean ± SEM. (**c**) Immunofluorescent imaging of AM isolated from 6-week-old WT and *Nkrp1b*$^{-/-}$ with Rab7 (red) and Lamp1 (green) specific labeling. Nuclei are shown in blue. Images are representative of (*n* = 3) for each genotype. Scale bar represents 20 µm. (**d**) Violin plot showing the quantification of colocalization between Rab7 and Lamp1 immunofluorescent signals shown in (S5c). Pearson's

correlation coefficient was used as a metric of colocalization. Statistical significance was assessed via unpaired, two-tailed Student's *t*-test. In (a, b, d) WT is indicated in blue and *Nkrp1b*$^{-/-}$ in orange.

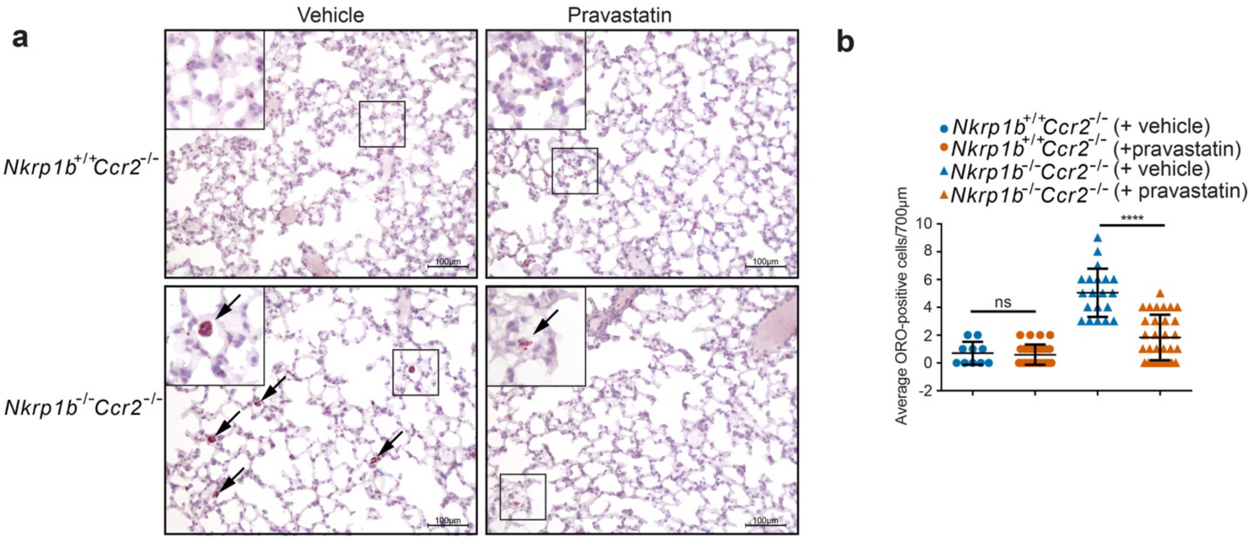

Supplementary Figure 6. In vivo statin administration rescues Nkrp1b-/- AM lipid accumulation. (**a**) ORO-stained frozen lung section of 5-week-old *Nkrp1b*$^{+/+}$*Ccr2*$^{-/-}$ and *Nkrp1b*$^{-/-}$*Ccr2*$^{-/-}$ mice after a 2-week treatment with water supplemented with vehicle or pravastatin. Images are representative of 2 independent experiments. Arrows indicate ORO positive cells. (**b**) Quantifications of ORO positive cells/μm^2 in frozen lung sections stained with ORO as seen in (a) from *Nkrp1b*$^{+/+}$*Ccr2*$^{-/-}$ (blue) and *Nkrp1b*$^{-/-}$*Ccr2*$^{-/-}$ (orange) mice after a 2-week treatment with water supplemented with vehicle or pravastatin. (n = 3 independent experiments) Data presented as mean ± SEM. Significance was determined by ordinary one-way ANOVA analysis $p < 0.0001$ with Sidak's correction for multiple comparisons where ****$p < 0.0001$. WT is indicated in blue and *Nkrp1b*$^{-/-}$ in orange. Source data are provided as a Source Data file.

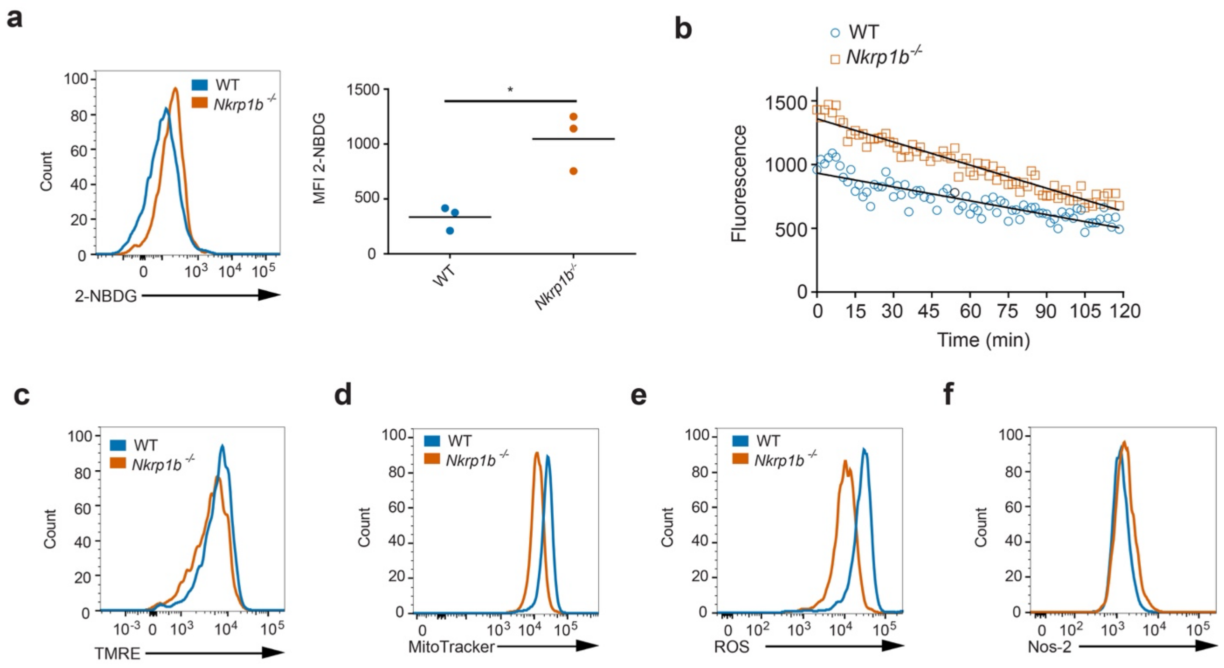

Supplementary Figure 7. *Nkrp1b$^{-/-}$* AM exhibit altered metabolic phenotype. (**a**) Fluorescent glucose uptake in AM isolated from WT and *Nkrp1b$^{-/-}$* mice cultured with 2-NBDG for 30 minutes. Plot representative of 3 different experiments. MFI of 2-NBDG glucose uptake between WT and *Nkrp1b$^{-/-}$* AM. Data presented as mean ± SEM. Statistics carried out via an unpaired, two-tailed Student's *t*-test where $^*p = 0.012$ ($n = 3$ mice per genotype). (**b**) Oxygen consumption assay of AM derived from 2-week-old WT and *Nkrp1b$^{-/-}$* mice as determined by release of oxygen reactive dye, higher fluorescence being indicative of higher O2 intake/consumption. Fluorescent measurements of oxygen consumption were taken every 90 seconds for 2 hours with fluorescence plotted over time. Comparison of fits analysis shows that lines of best fit for WT and *Nkrp1b$^{-/-}$* O2 consumption. Statistical significance was determined by linear regression analysis where p = 0.0001. (n = 3 for each genotype). (**c**) Representative histograms of AM isolated from 2-week-old WT and *Nkrp1b$^{-/-}$* mice showing levels of TMRE. Histograms are representative of 2 independent experiments. (**d**) Representative histograms of AM isolated from 2-week-old WT and *Nkrp1b$^{-/-}$*

mice showing levels of mito-tracker dye absorption. Histograms are representative of 2 independent experiments. (**e**) Representative histograms of AM isolated from 2-week-old WT and *Nkrp1b*$^{-/-}$ mice showing levels of ROS levels. Histograms are representative of 2 independent experiments. (**f**) Representative histograms of AM isolated from 2-week-old WT and *Nkrp1b*$^{-/-}$ mice showing levels of the amount of Nos-2 protein present. Histograms are representative of 2 independent experiments. In all graphs and histograms, WT is indicated in blue and *Nkrp1b*$^{-/-}$ in orange. Source data are provided as a Source Data file.

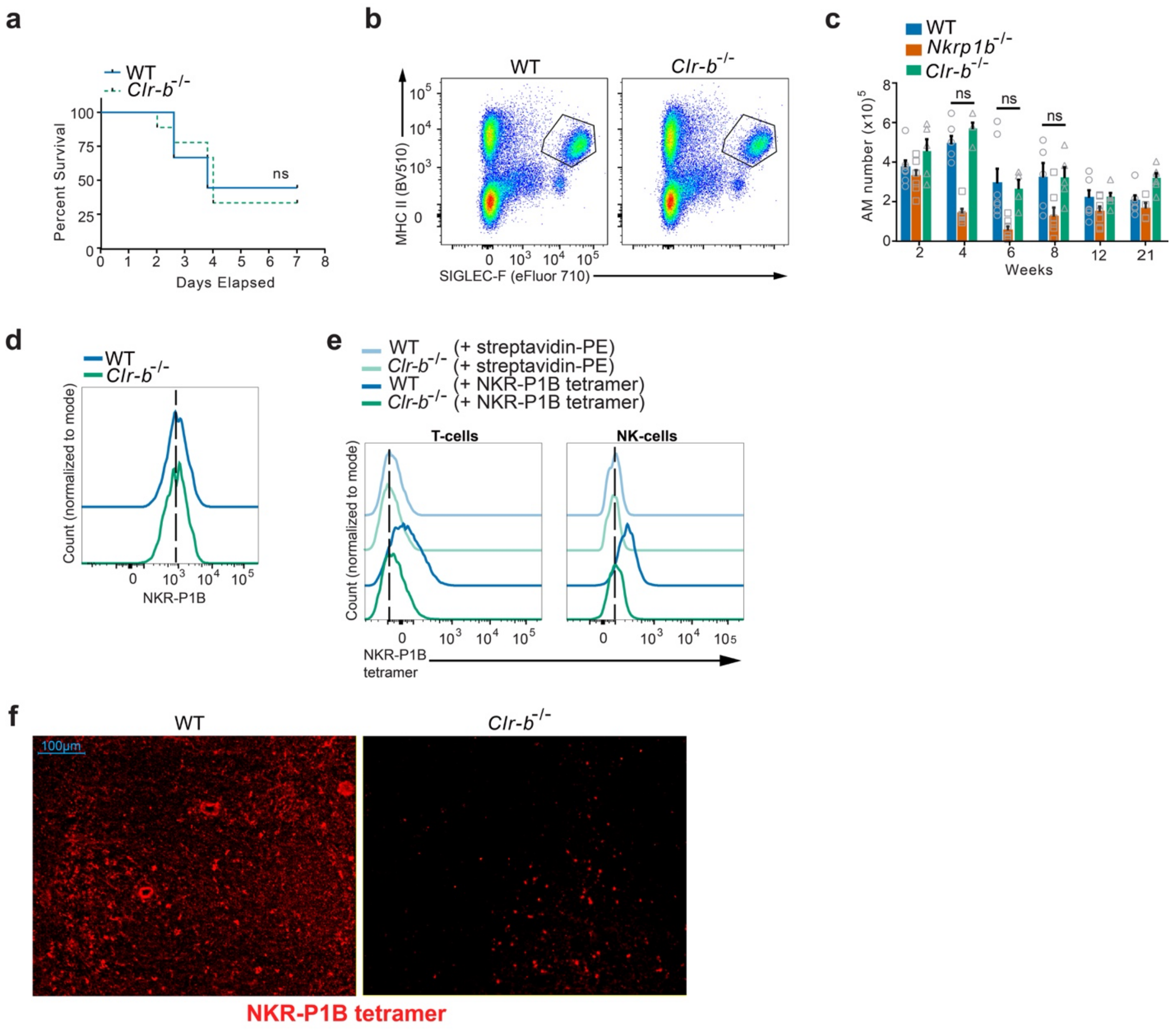

Supplementary Figure 8. Clr-b is dispensable for survival to lung pneumococcal infection but required for NKR-P1B tetramer staining of splenic tissue. (**a**) Kaplan-Meyer curves of WT versus *Clr-b*$^{-/-}$ mice susceptibility to *S. pneumoniae* at 6 weeks of age. *n* = 9 mice per genotype. Significance was determined by two-way ANOVA analysis $p < 0.0001$ with Sidak's correction for multiple comparisons. (**b**) Representative flow cytometry dot plots of AM found in the lungs of WT and *Clr-b*$^{-/-}$ mice at 6 weeks of age. Images are representative of 5 different experiments. (**c**) Quantifications of AM obtained by flow cytometric analysis from WT and *Clr-b*$^{-/-}$ mice at 2, 4, 6, 8, 12, and 21 weeks of age (*n* = 5, 6, 4, 5, and 12 mice per age, respectively). Data presented as

mean ± SEM. Statistical significance was determined by two-way ANOVA with Tukey's correction. (**d**) Histogram of NKR-P1B surface protein expression on WT and *Clr-b*$^{-/-}$ AM, representative of 3 independent experiments. (**e**) Expression of Clr-b on T-cells and NK cells obtained from spleens of WT and *Clr-b*$^{-/-}$ mice as determined by NKR-P1B tetramer binding. Histograms are representative of 3 independent experiments. (**f**) Confocal images of frozen spleen sections obtained from WT and *Clr-b*$^{-/-}$ mice stained with NKR-P1B tetramers (red) at 10x magnification. Images are representative of 3 independent experiments. In all graphs and histograms, WT is indicated in blue, *Nkrp1b*$^{-/-}$ in orange, and *Clr-b*$^{-/-}$ green. Source data are provided as a Source Data file.

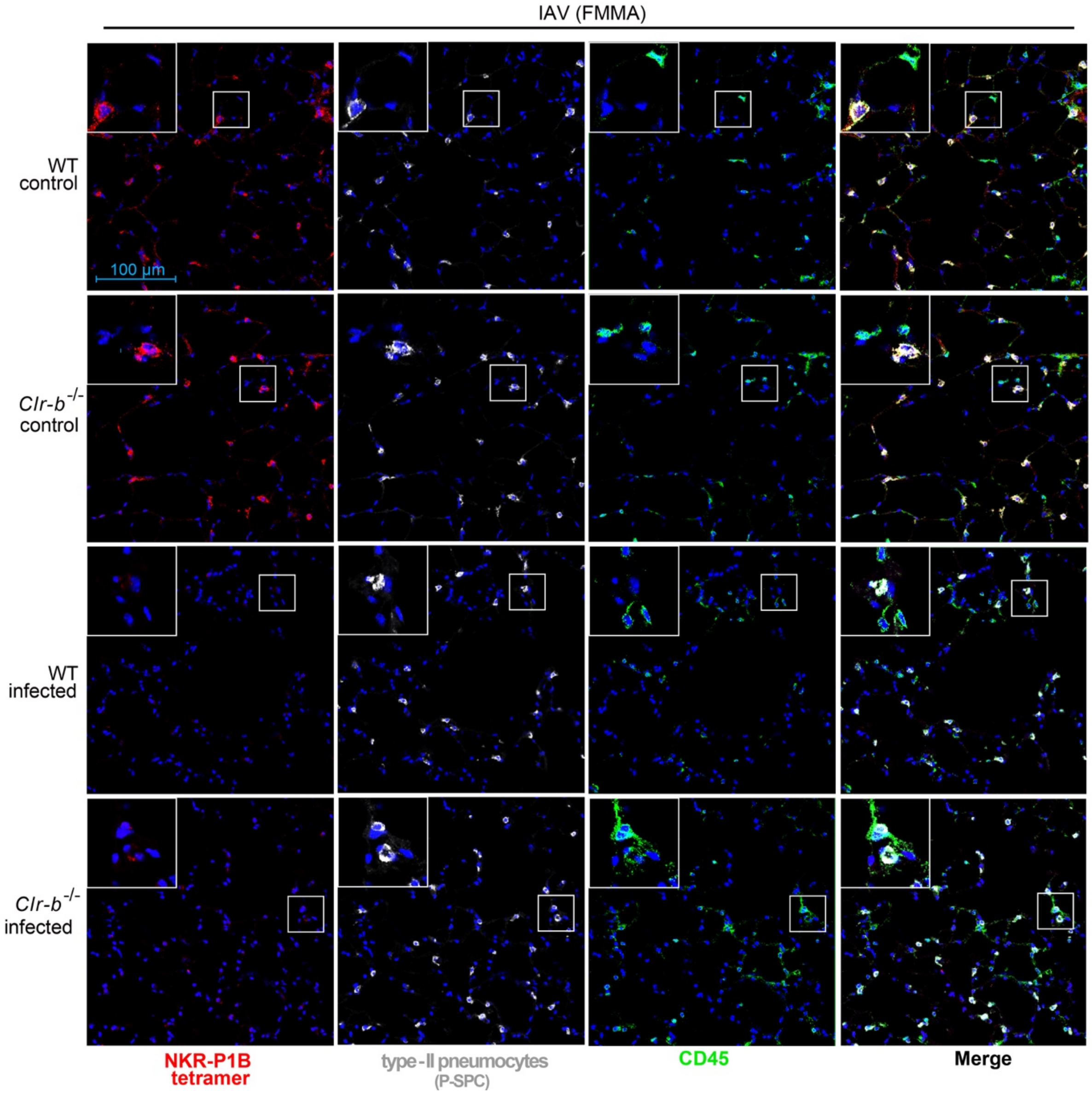

Supplementary Figure 9. Influenza A virus infection downregulates NKR-P1B ligand expression. Confocal image stacks of WT and *Clr-b*$^{-/-}$ frozen lung sections post-infection with IAV (FMMA) harvested 6-days post-infection respectively. Sections are stained with CD45 (Green), NKR-P1B tetramer (Red), DAPI (Blue) and pro-surfactant protein C (White). Data presented are representative of (*n* = 3 independent experiments).

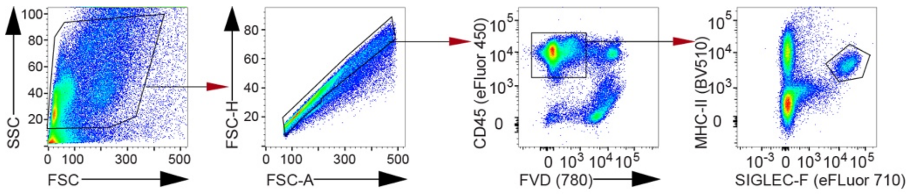

Supplementary Figure 10. Gating strategy for isolating AM from total mouse lung via flow cytometric phenotyping.
