# Supplementary material for: Alveolar macrophage metabolic programming via a C-type lectin receptor protects against lipo-toxicity and cell death
Source: Nat Commun. 2022 Nov 25;13:7272. doi: 10.1038/s41467-022-34935-w (PMC9700784; doi:10.1038/s41467-022-34935-w)
Supplement: Supplementary file 2 — Description of Additional Supplementary Files [file 41467_2022_34935_MOESM2_ESM.pdf]

Description of Additional Supplementary Files

File name: Supplementary Data 1

Description: Sequences for primers used in the quantification *CD36*, *CD63*, *Ldlr*, *Nceh*, *ABCA1*, *ABCG1*, *Serpinb6*, *Rara*, *Fabp5*, *ApoE*, and *Psap* transcript expression by RT-qPCR.
