# Supplementary material for: Alveolar macrophage metabolic programming via a C-type lectin receptor protects against lipo-toxicity and cell death
Source: Nat Commun. 2022 Nov 25;13:7272. doi: 10.1038/s41467-022-34935-w (PMC9700784; doi:10.1038/s41467-022-34935-w)
Supplement: Supplementary file 4 — Reporting Summary [file 41467_2022_34935_MOESM4_ESM.pdf]

nature portfolio

Corresponding author(s): Andrew Makrigiannis

Last updated by author(s): Oct 3, 2022

Reporting Summary

Nature Portfolio wishes to improve the reproducibility of the work that we publish. This form provides structure for consistency and transparency in reporting. For further information on Nature Portfolio policies, see our Editorial Policies and the Editorial Policy Checklist.

Statistics

For all statistical analyses, confirm that the following items are present in the figure legend, table legend, main text, or Methods section.

n/a	Confirmed	
☐	☒	The exact sample size (*n*) for each experimental group/condition, given as a discrete number and unit of measurement
☐	☒	A statement on whether measurements were taken from distinct samples or whether the same sample was measured repeatedly
☐	☒	The statistical test(s) used AND whether they are one- or two-sided<br>*Only common tests should be described solely by name; describe more complex techniques in the Methods section.*
☐	☒	A description of all covariates tested
☐	☒	A description of any assumptions or corrections, such as tests of normality and adjustment for multiple comparisons
☐	☒	A full description of the statistical parameters including central tendency (e.g. means) or other basic estimates (e.g. regression coefficient) AND variation (e.g. standard deviation) or associated estimates of uncertainty (e.g. confidence intervals)
☐	☒	For null hypothesis testing, the test statistic (e.g. *F*, *t*, *r*) with confidence intervals, effect sizes, degrees of freedom and *P* value noted<br>*Give P values as exact values whenever suitable.*
☒	☐	For Bayesian analysis, information on the choice of priors and Markov chain Monte Carlo settings
☒	☐	For hierarchical and complex designs, identification of the appropriate level for tests and full reporting of outcomes
☐	☒	Estimates of effect sizes (e.g. Cohen's *d*, Pearson's *r*), indicating how they were calculated

Our web collection on statistics for biologists contains articles on many of the points above.

Software and code

Policy information about availability of computer code

Data collection	Flow cytometry data were collected on a BD-LSR Fortessa cytometer<br>Microscopy data were collected on a Leica SP8 confocal microscope running Leica LasX Core acquisition software.<br>Leica Aperio slide scanner.<br>CLARIOstar High Performance Plate Reader (BMG LABTECH)<br>RT-qPCR data were collected on a Bio-Rad CFX96 Real-Time PCR analyzer.<br>RT-PCR gels and chemokine dotplots were imaged on a Bio-Rad ChemiDoc MP imaging system.<br>TEM micrographs were acquired on an JEOL 1230 TEM Transmission Electron Microscope with a Hamamatsu ORCA-HR digital camera
Data analysis	FlowJo 10.0.6 was used to analyze FACS data; GraphPad Prism v9 was used to generate plots and graphs; ImageJ v1.53 and ImageJ Coloc2 plugin and Leica LasX v3.7.6 analysis software were used to analyze microscopy data. RNA-Seq data was processed using Trimmomatic v0.39 and aligned using Cuffdiff v2.2.1 via the Galaxy web platform (https://usegalaxy.org).

For manuscripts utilizing custom algorithms or software that are central to the research but not yet described in published literature, software must be made available to editors and reviewers. We strongly encourage code deposition in a community repository (e.g. GitHub). See the Nature Portfolio guidelines for submitting code & software for further information.

Data

Policy information about availability of data

All manuscripts must include a data availability statement. This statement should provide the following information, where applicable:

- Accession codes, unique identifiers, or web links for publicly available datasets
- A description of any restrictions on data availability
- For clinical datasets or third party data, please ensure that the statement adheres to our policy

The RNA-seq datasets generated in this study have been deposited in the NCBI's Gene Expression Omnibus database under accession code GSE214867. [https://www.ncbi.nlm.nih.gov/geo/query/acc.cgi?acc=GSE214867]. Data generated in this study are provided in the Source Data file.

Human research participants

Policy information about studies involving human research participants and Sex and Gender in Research.

Reporting on sex and gender	*Use the terms sex (biological attribute) and gender (shaped by social and cultural circumstances) carefully in order to avoid confusing both terms. Indicate if findings apply to only one sex or gender; describe whether sex and gender were considered in study design whether sex and/or gender was determined based on self-reporting or assigned and methods used. Provide in the source data disaggregated sex and gender data where this information has been collected, and consent has been obtained for sharing of individual-level data; provide overall numbers in this Reporting Summary. Please state if this information has not been collected. Report sex- and gender-based analyses where performed, justify reasons for lack of sex- and gender-based analysis.*
Population characteristics	*Describe the covariate-relevant population characteristics of the human research participants (e.g. age, genotypic information, past and current diagnosis and treatment categories). If you filled out the behavioural & social sciences study design questions and have nothing to add here, write "See above."*
Recruitment	*Describe how participants were recruited. Outline any potential self-selection bias or other biases that may be present and how these are likely to impact results.*
Ethics oversight	*Identify the organization(s) that approved the study protocol.*

Note that full information on the approval of the study protocol must also be provided in the manuscript.

Field-specific reporting

Please select the one below that is the best fit for your research. If you are not sure, read the appropriate sections before making your selection.

[x] Life sciences [ ] Behavioural & social sciences [ ] Ecological, evolutionary & environmental sciences

For a reference copy of the document with all sections, see nature.com/documents/nr-reporting-summary-flat.pdf

Life sciences study design

All studies must disclose on these points even when the disclosure is negative.

Sample size	No a priori sample size determination was performed. Preliminary experiments were performed to estimate variances in each assay and determined sufficient sample size. The biological sample size is presented in the figure or stated in the figure legends section.
Data exclusions	No data exclusions
Replication	All attempts at replication were successful. The experiments number is stated in the figure legends.
Randomization	For in vitro experiments cells were isolated and randomly assigned to treatment or control groups. For in vivo mice experiments, age-matched mice were grouped randomly according to the genotype and sex and littermates were used where applicable.
Blinding	Blinding was used for pathological interpretations of histological sections and for experiments involving quantification of histological sections to prevent bias. Blinding was not used for any other experiments since the quantitative outputs of the experiment precludes any investigator bias.

Reporting for specific materials, systems and methods

We require information from authors about some types of materials, experimental systems and methods used in many studies. Here, indicate whether each material, system or method listed is relevant to your study. If you are not sure if a list item applies to your research, read the appropriate section before selecting a response.

Materials & experimental systems

n/a	Involved in the study
☐	☒ Antibodies
☐	☒ Eukaryotic cell lines
☒	☐ Palaeontology and archaeology
☐	☒ Animals and other organisms
☒	☐ Clinical data
☒	☐ Dual use research of concern

Methods

n/a	Involved in the study
☒	☐ ChIP-seq
☐	☒ Flow cytometry
☒	☐ MRI-based neuroimaging

Antibodies

Antibodies used

No. Antibody Name | Supplier | Catalog number | Clone name | Dilution
1.) anti-CD45-APC | BioLegend | 103112 | 30-F11 | 1:200
2.) anti-MHCII-BV510 | BioLegend | 107635 | M5/114.15.2 | 1:200
3.) anti-SIGLEC-F-PerCP-eFluor710 | Invitrogen | 46-1702-82 | 1RNM44N | 1:200
4.) anti-CD11c-FITC | eBioscience | 11-0114-85 | N418 | 1:200
5.) anti-SIGLEC-F | Invitrogen | 14-1702-82 | 1RNM44N | 1:200
6.) anti-PE | BioLegend | 408101 | PE001 | 1:200
7.) anti-rat IgG-AlexaFluor488 | Invitrogen | A-11001 | Polyclonal | 1:200
8.) anti-mouse IgG-AlexaFluor555 | BioLegend | 405324 | Poly4053 | 1:200
9.) anti-goat IgG-Cy5 | Invitrogen | A-1053 | Polyclonal | 1:200
10.) anti-CD45-FITC | BioLegend | 103101 | 30-F11 | 1:100
11.) anti-PSPTC | Abcam | ab90716 | Polyclonal | 1:500
12.) anti-NKRP1B (Clone 2D12) | Iizuka et al., 2003 | n/a | 2D12 | 1:100
13.) anti-Clrb (clone 4A6) | Carlyle et al., 2004 | n/a | 4A6 | 1:100
14.) anti-CD31-FITC | BioLegend | 102405 | 390 | 1:200
15.) anti-CD326 (EpCAM)-PE | BioLegend | 118205 | G8.8 | 1:200
16.) anti-CD104-FITC | BioLegend | 123605 | 346-11A | 1:200
17.) anti-GLUT1-AlexaFluor 405 | Abcam | ab210438 | EPR3915 | 1:25
18.) anti-G6PD-CoraLite 488 | Proteintech | CL488-66373 | 2A7B12 | 1:25
19.) anti-HK1-AlexaFluor647 | Abcam | ab197864 | EPR10134(B) | 1:25
20.) anti-CPT1A-AlexaFluor488 | Abcam | ab171449 | 8F6AE9 | 1:25
21.) anti-ACC1-AlexaFluor488 | Abcam | ab203994 | EPR4971 | 1:25
22.) anti-IDH2 PE | Abcam | ab212122 | EPR7577 | 1:25
23.) anti-ATP5A-AlexaFluor594 | Abcam | ab216385 | EPR13030(B) | 1:25
24.) anti-ASS1-PE | Abcam | ab210451 | EPR12398 | 1:25
25.) anti-PRP-PE | Abcam | ab197536 | EPR5154 | 1:25
26.) anti-pSTAT5-PE | Invitrogen | 12-9010-42 | SRBCZX | 1:50
27.) IgG1,k-PE Isotype Control | eBioscience | 12-4714-71 | P3.6.2.8.1 | 1:100
28.) anti-puromycin 647 | MilliporeSigma | MABE343-AF647 | 12D10 | 1:50
29.) IgG2a, k-AlexaFluor647 IsotypeControl | BD | 558053 | MOPC-173 | 1:100
30.) anti-phospho-p38MAPK | BioLegend | 690203 | A16016A | 1:50
31.) anti-NOS2 | BioLegend | 696805 | W16030C | 1:50
32.) anti-Rab7 | Abcam | ab137029 | EPR7589 | 1:50
33.) anti-CD107a/Lamp-1 | Biotechne | MAB4320-SP | 747203 | 1:50
34.) anti-NK1.1 | ATCC | HB-191 | PK136 | 100-200 ug i.p. inj.

Validation

Most antibodies used in this study are commercial (please see details above). NKR-P1B and Clr-b antibodies have been validated in the references indicated in the article.
1.) CD45,product citation is 136 from the manufacturer's website: : https://www.biolegend.com/en-us/products/apc-anti-mouse-cd45-antibody-97
2.) MHCII,product citation is 38 from the manufacturer's website: : https://www.biolegend.com/en-us/products/brilliant-violet-510-anti-mouse-i-a-i-e-antibody-7997
3.) SIGLEC,product citation is 8 from the manufacturer's website: : https://www.thermofisher.com/antibody/product/CD170-Siglec-F-Antibody-clone-1RNM44N-Monoclonal/46-1702-82
4.) CD11c,product citation is 205 from the manufacturer's website: : https://www.thermofisher.com/antibody/product/CD11c-Antibody-clone-N418-Monoclonal/11-0114-82
5.) SIGLEC,product citation is 6 from the manufacturer's website: : https://www.thermofisher.com/antibody/product/CD170-Siglec-F-Antibody-clone-1RNM44N-Monoclonal/14-1702-82
6.) PE,product citation is 11 from the manufacturer's website: : https://www.biolegend.com/en-us/products/purified-anti-phycoerythrin-pe-2813
7.) rat IgG,product citation is 6109 from the manufacturer's website: : https://www.thermofisher.com/antibody/product/Goat-anti-Mouse-IgG-H-L-Cross-Adsorbed-Secondary-Antibody-Polyclonal/A-11001

8.) mouse IgG,product citation is 6 from the manufacturer's website: : https://www.biolegend.com/en-us/products/alexa-fluor-555-goat-anti-mouse-igg-minimal-x-reactivity-9415
9.) goat IgG,product citation is 136 from the manufacturer's website: : https://www.thermofisher.com/antibody/product/Goat-anti-Rabbit-IgG-H-L-Cross-Adsorbed-Secondary-Antibody-Polyclonal/A10523
10.) CD45,product citation is 103 from the manufacturer's website: : https://www.biolegend.com/en-us/products/purified-anti-mouse-cd45-antibody-102
11.) PSPTC,product citation is 54 from the manufacturer's website: : https://www.abcam.com/prosurfactant-protein-c-antibody-ab90716.html?productWallTab=ShowAll
12.) NKRP1B,antibody is validated as referenced in the artcle [30]:
13.) Clrb,antibody is validated as referenced in the artcle [28]:
14.) CD31,product citation is 20 from the manufacturer's website: : https://www.biolegend.com/en-us/products/fitc-anti-mouse-cd31-antibody-120
15.) CD326,product citation is 23 from the manufacturer's website: : https://www.biolegend.com/en-us/products/pe-anti-mouse-cd326-ep-cam-antibody-4726
16.) CD104,product citation is 2 from the manufacturer's website: : https://www.biolegend.com/en-us/products/fitc-anti-mouse-cd104-antibody-4491
17.) GLUT1,product gave a positive signal in mouse CD4 Tcells, BMDMs, brain cells: https://www.abcam.com/alexa-fluor-405-glucose-transporter-glut1-antibody-epr3915-ab210438.html?productWallTab=ShowAll
18.) G6PD,product shows reactivity with human, mouse samples: https://www.ptglab.com/products/G6PD-Antibody-CL488-66373.htm
19.) HK1,product gave a positive signal in HCT116 cells: https://www.abcam.com/alexa-fluor-647-hexokinase-1-antibody-epr10134b-mitochondrial-outer-membrane-marker-ab197864.html?productWallTab=ShowAll
20.) CPT1A,product citation is 8 from the manufacturer's website: : https://www.abcam.com/alexa-fluor-488-cpt1a-antibody-8f6ae9-ab171449.html?productWallTab=ShowAll
21.) ACC1,product gave a positive signal in HepG2 cells: https://www.abcam.com/alexa-fluor-488-acetyl-coenzyme-a-carboxylase-antibody-epr4971-ab203994.html?productWallTab=ShowAll
22.) IDH2 PE,product has been verified in ICC/IF: https://www.abcam.com/pe-idh2-antibody-epr7577-ab212122.html?productWallTab=ShowAll
23.) ATP5A,product citation is 1 from the manufacturer's website: : https://www.abcam.com/alexa-fluor-594-atp5a-antibody-epr13030b-ab216385.html?productWallTab=ShowAll
24.) ASS1,product gave a positive signal in HeLa & mouse CD8 Tcells: https://www.abcam.com/pe-ass1-antibody-epr12398-ab210451.html?productWallTab=ShowAll
25.) PRP,product gave a positive signal in Hek293 cells: https://www.abcam.com/alexa-fluor-488-peroxiredoxin-2prp-antibody-epr5154-ab197536.html
26.) pSTAT5,product citation is 15 from the manufacturer's website: : https://www.thermofisher.com/antibody/product/Phospho-STAT5-Tyr694-Antibody-clone-SRBCZX-Monoclonal/12-9010-42
27.) IgG1,k,product citation is 144 from the manufacturer's website: : https://www.thermofisher.com/antibody/product/Mouse-IgG1-kappa-clone-P3-6-2-8-1-Isotype-Control/12-4714-42
28.) puromycin 647,product detected puromycin-incorporated neosynthesized proteins in puromycin treated HeLa cells: https://www.emdmillipore.com/CA/en/product/Anti-Puromycin-clone-12D10-Alexa-Fluor-647-Conjugate-Antibody,MM_NF-MABE343-AF647?ReferrerURL=https%3A%2F%2Fwww.google.com%2F
29.) IgG2a, k,product citation is 1 from the manufacturer's website: : https://www.bdbiosciences.com/en-eu/products/reagents/flow-cytometry-reagents/research-reagents/single-color-antibodies-ruo/alexa-fluor-647-mouse-igg2a-isotype-control.558053
30.) phospho-p38MAPK,product is "quality control tested by [ICFC]": https://www.biolegend.com/en-us/products/pe-anti-p38-mapk-phospho-thr180-tyr182-antibody-18747
31.) NOS2,product is "quality control tested by [ICFC]": https://www.biolegend.com/en-us/products/pe-anti-nos2-inos-antibody-19910
32.) Rab7,product citation is 69 from the manufacturer's website: : https://www.abcam.com/rab7-antibody-epr7589-ab137029.html?productWallTab=ShowAll
33.) CD107a,product citation is 2 from the manufacturer's website: : https://www.bio-techne.com/p/antibodies/mouse-lamp1-cd107a-antibody-747203_mab4320
34.) NK1.1,product citation is 69 from the manufacturer's website: : https://www.atcc.org/products/hb-191

Eukaryotic cell lines

Policy information about cell lines and Sex and Gender in Research

Cell line source(s)	The mouse cell line MLE-12 (CRL-2110) and the human cell line HEK293T(CRL3216) were purchased from ATCC.
Authentication	None of the cell lines were authenticated.
Mycoplasma contamination	Cell lines were not tested for mycoplasma contamination
Commonly misidentified lines (See ICLAC register)	None to declare

Animals and other research organisms

Policy information about studies involving animals; ARRIVE guidelines recommended for reporting animal research, and Sex and Gender in Research

Laboratory animals	Animals were housed under specific pathogen-free conditions. All mice were maintained under a 12 h light/12 h dark cycle with free access to food and water in a temperature (22±1°C) and humidity (52±1%) controlled room.<br>C57BL/6 (WT) mice: ages 2-21 weeks old, age and sex matched, both males and females used<br>NKR-P1B deficient mice on C57BL/6 background: 2-21 weeks old, age and sex matched, both males and females used<br>CCR2 and NKR-P1B double deficient mice on C57BL/6 background: 2-21 weeks old, age and sex matched, both males and females used<br>Clr-b deficient mice on C57BL/6 background: 2-21 weeks old, age and sex matched, both males and females used
Wild animals	Study did not involve wild animals
Reporting on sex	Findings reported in this study apply to both sexes as study design and experimental setups included both, male and female animals.
Field-collected samples	Study did not involved field-collected samples
Ethics oversight	All animal experiments were conducted in accordance with protocols 19-135 and 20-084 approved by the Dalhousie University Animal Care Committee and the Canadian Council on the Use of Laboratory Animals.

Note that full information on the approval of the study protocol must also be provided in the manuscript.

Flow Cytometry

Plots

Confirm that:

- [x] The axis labels state the marker and fluorochrome used (e.g. CD4-FITC).
- [x] The axis scales are clearly visible. Include numbers along axes only for bottom left plot of group (a 'group' is an analysis of identical markers).
- [x] All plots are contour plots with outliers or pseudocolor plots.
- [x] A numerical value for number of cells or percentage (with statistics) is provided.

Methodology

Sample preparation	Mice were euthanized, lungs excised and homogenized with a razor blade. The homogenate was incubated at 37oC with 5% CO2 in the presence of 5mg/mL collagenase D (Worthington Biochemical Corporation CAT# LS004188) for 1 hour. The resulting homogenate was then passed through a 70 #m cell strainer (Falcon CAT# 352350). The cell suspension was then subjected to a 20% percoll (GE Healthcare CAT# 17-0891-01) gradient and spun at 2000xg for 30 minutes. The supernatant was pipetted off and ACK lysis was performed to lyse the red blood cells. The final cell suspension was resuspended in PBS, cell counts were determined and then surface stained for flow cytometry. Single cell lung suspensions were stained with anti-CD45-APC (BioLegend CAT# 103112), anti-MHCII-BV510 (BioLegend CAT# 107635), anti-SIGLEC-F-PerCP-eFluor 710 (Invitrogen CAT# 46-1702-82) and in some experiments with anti-CD11c-FITC (eBioscience CAT# 11-0114-85), anti-NKR-P1B (generous gift from Dr. Koho Iizuka, University of Minnesota) and anti-Clr-b antibodies is described previously 28. After gating out doublets, AMØ were identified as CD45+MHCIImidSIGLEC-F+ cells with high forward and side scatter characteristics. In lavage experiments, mice were anaesthetized by CO2 asphyxiation, the trachea exposed and then cannulated using a 20-gauge syringe wrapped in surgical tubing. Up to 1 mL of cold PBS was then flushed into lung, withdrawn and the lavage fluid placed into a sterile 15 mL tube. This was repeated 3 more times. The lavage AMØ were then used for downstream experiments. Cell isolation from the spleen was conducted by excising the spleen from the mouse and crushing it between two microscope slides. The resulting cell suspension was subjected to ACK lysis after which it was resuspended in 10 mL PBS and used for flow cytometry. For isolation of Kupffer cells, mouse livers were mashed through a 70 µm cell strainer, spun down, and resuspended in 37% percoll, and centrifuged at 700 g for 30 minutes. Afterward, the cells were subjected to ACK lysis, resuspended in 100 µL, and stained for flow cytometry. Large and small peritoneal macrophages were extracted by injecting cold PBS into the peritoneal cavity, gently massaging the area to dislodge any cells, and then removing the PBS into a tube. This process was repeated 4 times. Cells were then spun down, resuspended in 100 µL, and stained for flow cytometry
Instrument	Beckton-Dickinson LSR Fortessa SORP
Software	FlowJo v10.0.6
Cell population abundance	We analyzed 10,000 single cells which are live.
Gating strategy	Very low FSC and SSC event were excluded. Doublets were excluded based on FSC-A, FSC-H. CD45+ MHC-II-mid SIGLEC-F+ cells were identified as alveolar macrophages.

- [x] Tick this box to confirm that a figure exemplifying the gating strategy is provided in the Supplementary Information.
